# *Lycium barbarum*-Derived Extracellular Vesicles Ameliorate Myocardial Hypertrophy in Heart Failure with Preserved Ejection Fraction, Associated with Reduced Cardiomyocyte Apoptosis

**DOI:** 10.3390/nu18152564

**Published:** 2026-08-05

**Authors:** Wenhao Hao, Yuxin Zhao, Huimin Cui, Andong Liu, Wei Gong, Kangling Wang, Hong Lin, Lei Hao, Fanfan Han, Jianjun Yang

**Affiliations:** 1 School of Public Health, Ningxia Medical University, Yinchuan 750004, China; h04230518@163.com (W.H.);; 2 Ningxia Key Laboratory of Environmental Factors and Chronic Disease Control, Yinchuan 750004, China; 3 Department of Allied and Public Health, Indiana University of Pennsylvania, Indiana, PA 15705, USA

**Keywords:** heart failure with preserved ejection fraction, *Lycium barbarum*-derived extracellular vesicles, myocardial hypertrophy, apoptosis, *TP53*, mitochondrial function

## Abstract

Background: Non-invasive therapies for heart failure represent one of the current major focuses in clinical research. Plant-derived extracellular vesicles (PEVs) have recently emerged as a promising therapeutic modality. Objectives: In this study, we investigated the therapeutic potential of PEVs in heart failure with preserved ejection fraction (HFpEF), focusing on extracellular vesicles derived from *Lycium barbarum* (Gq-EVs). Methods: We first obtained Gq-EVs with high biological activity and high concentration from *Lycium barbarum* juice and characterized them by using transmission electron microscopy, nanoparticle tracking analysis, and metabolite composition analysis. Results: Our in vivo findings indicate that nebulized inhalation of Gq-EVs for 7, 14, and 21 days improved abnormal cardiac diastolic function, reduced excessive myocardial hypertrophy, and attenuated inflammatory cell infiltration and collagen deposition in HFpEF mice. Further in vivo and in vitro experiments showed that Gq-EVs can reach injured cardiac tissue, improve mitochondrial function, and are associated with reduced cardiomyocyte apoptosis and amelioration of myocardial hypertrophy, potentially involving *TP53*-related apoptotic signaling. Conclusions: Overall, nebulized delivery of Gq-EVs may be a relatively non-invasive, well-tolerated, and cost-effective therapeutic strategy for HFpEF, highlighting the potential of PEV-based nanotherapies and offering a sustainable approach for the medical utilization of *Lycium barbarum* resources.

## 1. Introduction

Heart failure with preserved ejection fraction (HFpEF) is mainly characterized by a relatively normal ejection fraction and impaired diastolic function of the heart [1]. HFpEF, which accounts for roughly half of all heart failure cases, is particularly common among older adults and individuals with multiple comorbidities [2,3,4]. Currently, the optimal pharmacological management of HFpEF focuses on sodium glucose-cotransporter 2 (SGLT2) inhibitors, mineralocorticoid receptor antagonists (MRA), incretin-based therapies, angiotensin receptor-endothelin receptor inhibitors, and angiotensin receptor blockers (ARBs) [5,6,7,8]. The use of these drugs often comes with side effects, such as SGLT2 posing a risk of mycotic infections, urinary tract infections, and increased urination, MRA causing hyperkalemia, and ARNIS leading to hypotension and renal dysfunction [9,10,11,12]. Therefore, based on the SGLT2 treatment strategy, we can explore some natural compound research as a complementary exploratory avenue within the broader context of ongoing therapeutic development. Safe, non-toxic, and low-cost natural compounds may target key mechanisms such as myocardial stiffness, inflammation, and endothelial dysfunction, offering a potentially effective strategy to improve outcomes in HFpEF.

As crucial nanocarriers for intercellular communication and biological delivery, plant-derived extracellular vesicles (PEVs) have garnered significant attention in recent years, with rich sources, high acquisition rates, low cost, high biological activity, and natural safety [13,14,15,16]. PEVs, containing a variety of proteins, nucleic acids, lipids, and small-molecule bioactive substances, exert multi-target effects, including anti-inflammatory [17,18,19], antioxidant activities [20,21], anti-apoptosis [22,23], and protective effects on the heart [24,25,26], and are emerging as a new nanoscale therapeutic approach in cardiovascular treatment.

Among PEVs, those derived from *Lycium barbarum* (Gq-EVs) are particularly promising, as they are enriched with diverse bioactive components and possess intrinsic structural and size-related features that may enhance their therapeutic potential for HFpEF. Despite these advantages, the cardioprotective effects of Gq-EVs in HFpEF, especially their ability to alleviate myocardial hypertrophy and improve cardiac function, have not been systematically evaluated. Another study found that Lachnospiraceae-derived extracellular vesicles mediate the cardioprotective effects of barley leaf in myocardial infarction by improving intestinal stem cell function [27]. A stem cell-derived exosome nebulization therapy ameliorated cardiac repair by improving left ventricular function, reducing fibrotic tissue, and promoting cardiomyocyte proliferation [28]. When administered via nebulization, Mesenchymal stromal cell-derived extracellular vesicles from human umbilical cord (hUCMSC-EVs) predominantly accumulated in murine lungs and ameliorated bleomycin-induced pulmonary fibrosis, with increased survival rate (from 20% to 80%), restored lung volume, and attenuated injury severity accompanied by elevated oxyhemoglobin saturation and improved pulmonary function evaluations [29]. Our study found that the angiotensin-converting enzyme inhibitory peptides extracted from *Lycium barbarum* can alleviate cardiac hypertrophy in HFpEF mice related to the ACE2/Ang (1–7)/MasR axis and the PKCβ1 pathway [30]. We also utilized molecular docking and network pharmacology methods to identify potential targets of ACE-inhibitory peptides derived from the residue of *Lycium barbarum* related to HFpEF [31]. Gq-EVs extracted from *Lycium barbarum* could inhibit dexamethasone-induced muscle atrophy by the AMPK-SIRT1-PGC1α signaling pathway [32], reduce Abeta-induced apoptosis in PC12 cells through the MAPK and PI3K/AKT signaling pathways [22], promote spinal cord injury repair [33], and enhance skin barrier repair [34].

Furthermore, with advancements in drug delivery technology, the transition from traditional intravenous injection to aerosol inhalation has been achieved, with the latter emerging as a specific and highly efficient route of administration [35,36,37,38]. Nebulization delivery enables the therapeutic agent to deposit directly in the lungs and enter the pulmonary circulation, maximizing local concentration while minimizing systemic adverse effects. This method has demonstrated potential to improve therapeutic outcomes and enhance patient compliance in the management of HFpEF. A recent study reported an innovative and non-invasive approach called stem cell-derived exosome nebulization therapy [38]. Delivering exosomes from pulmonary globular cells to the heart by inhalation provides a new avenue for regenerative applications.

In this study, we established a quality-controlled protocol for the preparation of Gq-EVs and employed non-invasive inhalation delivery to evaluate their efficacy and safety in a murine HFpEF model, as well as to explore the underlying mechanisms.

## 2. Materials and Methods

### 2.1. Gq-EVs Preparation

*Lycium barbarum* juice was provided by Beryl Wolfberry Limited by Share Ltd. (Yinchuan, China). The juice was first centrifuged at 3000× *g* for 20 min and 10,000× *g* for 60 min, then ultracentrifuged at 150,000× *g* for 1.5 h. The suspension of Gq-EVs was transferred to a discontinuous sucrose gradient (8%/30%/45%/60%) and ultracentrifuged again at 150,000× *g* for an additional 1.5 h [29]. The visible band between 30% and 45% layers was harvested. The suspension of Gq-EVs was stored at 4 °C for short-term use and at −80 °C for long-term storage. Morphological observation of Gq-EVs was measured using transmission electron microscopy (HT-7700, Hitachi, Tokyo, Japan). Size and concentration were determined by nanoparticle tracking analysis (NTA) using the Nanosight NS300 (Malvern Panalytical, Malvern, UK). The stability of Gq-EVs was evaluated by measuring zeta potential using a Malvern Zetasizer Nano ZS90 (Malvern Panalytical, UK).

### 2.2. Animal Housing

Seven-week-old male C57BL/6J mice were purchased from Charles River Laboratories (Beijing, China). All mice were housed in the Experimental Animal Center of Ningxia Medical University (license number: SYXK Ning. 2020–0001) under controlled conditions of 20–25 °C, 40–50% humidity, and a 12 h/12 h light/dark cycle with ad libitum access to water and food for acclimation for 1 week before the experiment. All experimental procedures were approved by the Institutional Ethics Committee of Ningxia Medical University (IACUC–NYLAC–2024–212).

### 2.3. Induction of Mouse Model of HFpEF

To establish HFpEF, the 8-week-old mice were randomly assigned to two groups (*n* = 50). The control (Ctrl) group, which received normal chow (Jiangsu Xietong Pharmaceutical Bioengineering Co., Ltd., Nanjing, China) and regular drinking water (*n* = 10). To establish the HFpEF model, mice were fed a high-fat diet (60 kcal% from fat, D12492, Research Diet Inc, New Brunswick, NJ, USA) and received 0.5 g/L N^ω^-Nitro-L-arginine methyl ester hydrochloride (N5751, Sigma-Aldrich, St. Louis, MO, USA) in the drinking water for 8 weeks (*n* = 40). Then, the HFpEF group mice were divided into the following groups (10 mice per group): HFpEF + PBS (treated with PBS as vehicle for 21 consecutive days), HFpEF + Gq-7 (treated with Gq-EVs inhalation through a nebulizer for 7 consecutive days), HFpEF + Gq-14 (treated with Gq-EVs inhalation through a nebulizer for 14 consecutive days) and HFpEF + Gq-21 (treated with Gq-EVs inhalation through a nebulizer for 21 consecutive days). The dose of Gq-EVs administered to mice in the HFpEF + Gq-7, HFpEF + Gq-14, and HFpEF + Gq-21 groups was 1 × 10^11^ particles/kg, as referred to in the literature on exosome aerosol inhalation interventions [28].

During the experiment, body weight, blood glucose levels, glucose tolerance, blood pressure, and cardiac function were monitored.

### 2.4. Non-Targeted Metabolomic Analysis

Non-targeted metabolomic analysis was evaluated by Wuhan Metware Biotechnology Co., Ltd. (Metware Biotechnology Co., Ltd., Wuhan, China). The unbiased detection of metabolite composition in Gq-EVs was performed using Ultra-Performance Liquid Chromatography-Mass Spectrometry (UPLC-MS/MS, AB SCIEX Pte. Ltd., Marlborough, MA, USA), followed by pathway analysis of differential metabolites via bioinformatics analysis. Based on the Metware database, secondary spectrum information was used to identify the substances. During the analysis, the isotope signals, the repeated signals of K+, Na+, and NH4+, and the repeated signals of the fragments of other substances with higher molecular weight were removed, thereby improving the accuracy and reliability of the identification results.

### 2.5. UPLC-MS/MS

The samples were vacuum freeze-dried and dissolved in 70% methanol for UPLC-MS/MS analysis. The data acquisition system comprised UPLC-MS/MS. Metabolite quantification was performed using triple quadrupole mass spectrometry (Shimadzu Corporation, Tokyo, Japan) with multiple reaction monitoring. The liquid chromatography conditions were as follows. Chromatography column: Agilent SB-C18 (Agilent, St. Clara, CA, USA) 1.8 μm, 2.1 mm × 100 mm; Source mobile phase: Phase A was ultrapure water (0.1% formic acid added), and phase B was acetonitrile (0.1% formic acid added); Elution gradient: Phase B proportion increased linearly from 0 to 95% in 0.00 min, then maintained at 95% 1 min, 10.00–11.10 min, and decreased to 5% before reaching equilibrium at 14 min; Flow rate: 0.35 mL/min; Column temperature: 40 °C; Sample injection volume: 2 mL. The mass spectrometry setup features an electrospray ionization source at 500 °C, with ion spray voltages of 5500 V (positive mode) and −4500 V (negative mode). Gas ionization sources are set at 50, 60, and 25 psi, respectively, while collision-induced ionization parameters are configured at a high level.

### 2.6. Use of the TP53 Inhibitor Pifithrin-α (PFT-α) In Vivo

Eight-week-old mice were randomly assigned to two initial groups (*n* = 24 total). The control (Ctrl) group, which received normal chow and regular drinking water (*n* = 6). The HFpEF group mice were established by feeding a high-fat diet and administering 0.5 g/L N^ω^-Nitro-L-arginine methyl ester hydrochloride in drinking water for 8 weeks (*n* = 18). After HFpEF induction, mice were further divided into three treatment groups (6 mice per group): (1) HFpEF + PBS (treated with PBS as vehicle for 21 consecutive days); (2) HFpEF + Gq-21 (treated with Gq-EVs inhalation through a nebulizer for 21 consecutive days); and (3) HFpEF + PFT-α, the TP53 inhibitor (1.1 mg/kg/day, 21 consecutive days).

### 2.7. Tail-Cuff Blood Pressure Recordings

Blood pressure was measured by the tail-cuff method and blood pressure monitor (BP98A Series, Softron Biotechnology Co., Ltd., Tokyo, Japan) in the 8th (16-week-old mice, the HFpEF model was established), 9th (mice began to be treated with Gq-EVs), 10th, 11th, and 16th weeks (the end of the experiment). Recordings were performed under steady-state conditions until three stable continuous measurements were obtained.

### 2.8. Measurement of Cardiac Function

The cardiac function of mice was measured by an ultra-high-resolution small-animal ultrasound imaging system. Using three ultrasound modalities—B-mode ultrasound, M-mode ultrasound, and Doppler ultrasound—it was confirmed that the LVEF is ≥50%, and that structural abnormalities (left atrial enlargement, left ventricular hypertrophy) or diastolic dysfunction were identified. The interventricular septum thickness (IVS), left ventricular posterior wall thickness (LVPW), left ventricular diameter (LVD), ejection fraction (EF), and left ventricular mass (LV Mass) were obtained through long-axis M-mode scanning. The Apical four-chamber views were obtained at the mitral valve level by pulsed-wave Doppler imaging to measure the isovolumic relaxation time (IVRT), mitral inflow E-wave and A-wave.

### 2.9. Glucose Tolerance Test (GTT)

Mice were fasted for 6 h and then received an intraperitoneal injection of D-glucose solution (2 g/kg·body weight; Solarbio, Beijing, China). Blood glucose levels were measured 0, 30, 60, 90, and 120 min after injection.

### 2.10. In Vivo Imaging of Small Animals and Ex Vivo IVIS Imaging

Mixed Gq-EVs with DiR dye and incubated at room temperature for 30 min, followed by ultracentrifugation at 150,000× *g* for 1 h to obtain Gq-EVs-DiR. Mice were subjected to nebulized inhalation of Gq-EVs-DiR for 15 min. In vivo imaging was carried out at 0, 0.5 h, 1 h, 6 h, 12 h, and 24 h. Subsequently, the lung and heart tissues were observed by ex vivo IVIS imaging.

### 2.11. Biochemical Assays

After the experiment, blood samples were collected from mice to measure the serum levels of n-terminal pro-brain natriuretic peptide (NT-pro BNP) using enzyme-linked immunosorbent assay (ELISA) kits (Jiangsu Jingmei Biological Technology Co., Ltd., Yancheng, China), as well as Aspartate Aminotransferase (AST) and Alanine Aminotransferase (ALT) using ELISA kits (Beijing Puli Gene Technology Co., Ltd., Beijing, China).

### 2.12. Assessment of Cardiac Pathology

Hematoxylin and eosin (H&E) staining (Thermo Fisher Scientific, Shanghai, China) was used to evaluate the cardiac morphology and inflammatory cell infiltration. Cardiac collagen accumulation was assessed by Masson’s trichrome staining (Solarbio, Beijing, China). For the evaluation of cardiac hypertrophy, heart sections were stained with WGA (GeneTex, Irvine, CA, USA). ImageJ2 (National Institutes of Health, Bethesda, MD, USA) was used for quantification.

### 2.13. Immunofluorescence Staining

The sections were incubated with the primary antibody cTnT (1:200, Proteintech, Wuhan, China) and incubated the sections at 4 °C overnight. On the second day, after washing with PBS (Solarbio, Beijing, China), the sections were incubated with a secondary antibody (Alexa Fluor^®^ 488-conjugated Affinipure Goat Anti-Rabbit IgG; 1:500, Jackson ImmunoResearch Labs Cat# 111–545–003, RRID: AB_2338046, Philadelphia, PA, USA) for 1 h at room temperature. Then we washed the sections with PBS. The sections were mounted with an anti-fade mounting medium containing DAPI staining solution, and then observed under a fluorescence microscope. For frozen sections, the heart tissues were placed in optimal cutting temperature matrix compound at a temperature of −20 °C. The sections were equilibrated at room temperature for 15 min before performing antigen retrieval.

### 2.14. In Vitro Transcellular Transport of Gq-EVs

Alveolar epithelial cells A549 obtained from the Cancer Research Institute of Xi’an Jiaotong University were cultured in the upper chamber of the transwell chamber, and culture medium was added to the lower chamber. Gq-EVs were added to the upper chamber to culture for 24 h; the content of Gq-EVs in the culture medium of the lower chamber was detected to evaluate if Gq-EVs could traverse the pulmonary barrier in vitro.

### 2.15. Cell Culture and Treatments

To study the effect of Gq-EVs on apoptosis of cardiomyocytes, AC16 cells (cultured in cardiomyocyte-specific medium containing 10% fetal bovine serum) were treated with Gq-EVs for 24 h and were stimulated with palmitic acid (PA) for 24 h. Cell Counting Kit-8 (CCK-8, Beyotime, Shanghai, China) was used to detect cell viability. The cells were treated with different concentrations of Gq-EVs (5 × 10^7^, 1 × 10^8^, 5 × 10^8^, 1 × 10^9^, 5 × 10^9^, 1 × 10^10^ and 1 × 10^11^ particles/mL). Cells were stimulated with 20 μM PFT-α or 5 μM PFT-μ for 5h to inhibit *TP53*.

### 2.16. Quantitative Real-Time Polymerase Chain Reaction (qRT-PCR)

Cardiac and cellular RNA was extracted and analyzed by qRT-PCR analysis for determining the RNA levels, using primers for Bcl-2-associated X protein (*BAX*), B-cell lymphoma 2 (*BCL2*), brain natriuretic peptide (*Bnp*), brain natriuretic peptide (*BNP*), β-myosin heavy chain (*β-Mhc*), β-myosin heavy chain (*β-MHC*), Dynamin-related protein 1 (*DRP1*), Mitofusin 2 (*MFN2*), Optic atrophy 1 (*OPA1*), Peroxisome proliferator-activated receptor gamma coactivator 1-alpha (*PGC-1α*), Mitofusin 2 (*MFN2*), *Tp53* (Sangon Biotech, Shanghai, China). The target mRNA level was normalized to *Rplp0* mRNA. The procedures for RNA extraction and qRT-PCR were described previously [39]. The primer sequences were shown in Table S1.

### 2.17. Retrieval of Target Genes for Gq-EVs and HFpEF

Retrieve and obtain target genes using the keywords “Gouqi-derived nanovesicles”, “*Lycium barbarum*-derived nanovesicles”, “Wolfberry-derived nanovesicles” and “*Lycium barbarum* extracellular vesicles” through the CHEMBL (https://www.ebi.ac.uk/chembl, accessed on 13 April 2025) and Swiss Target Prediction (http://swisstargetprediction.ch/, accessed on 13 April 2025) databases. When querying target genes in the Swiss Target Prediction database, it is necessary to find the Canonical SMILES of the *Lycium barbarum* extracellular vesicles components in PubChem (https://pubchem.ncbi.nlm.nih.gov/, accessed on 13 April 2025), copy them to Swiss Target Prediction for target gene prediction. Finally, the target genes were standardized using the UniProt database, with species restricted to Homo sapiens and a probability threshold set at >0.9 (high confidence) to identify genes with drug-targeting potential.

Use “heart failure with preserved ejection fraction” as the keyword to screen disease target genes of HFpEF in the OMIM database (https://www.omim.org/, accessed on 13 April 2025), Gencards database (https://www.genecards.org/, accessed on 13 April 2025), and Dis Ge NET database (https://www.disgenet.org/, accessed on 13 April 2025), respectively. Finally, convert the obtained disease targets in the UniProt database and obtain disease target genes under the condition of “Homo sapiens”. After deduplication, a total of 1407 disease-related target genes associated with HFpEF were identified.

The 2342 “Gq-EVs predicted target sets” obtained were intersected with the 1407 “HFpEF disease target sets,” yielding 279 shared targets. These targets are considered core therapeutic targets for Gq-EVs in the intervention of HFpEF.

### 2.18. Construction of the Protein–Protein Interaction (PPI) Network

Target genes were input into STRING to generate a PPI network. The resulting PPI network was visualized using the Cytoscape 3.10 software. Topological parameters, such as the node degree (i.e., the number of other targets interacting with the target) and betweenness centrality (which measures the importance of the target as a bridge connecting different modules in the network), of each target, were calculated. The top 25 targets were selected for display based on the node degree and betweenness centrality.

### 2.19. Statistical Analysis

Data analysis and charting were performed using GraphPad Prism 9.0.0 (GraphPad Software, San Diego, CA, USA). For comparisons among three or more groups, one-way analysis of variance (one-way ANOVA) was used, followed by Tukey’s multiple comparison test for pairwise comparisons. Comparisons between two groups were performed using a two-tailed *t*-test. *p*-values less than 0.05 were considered statistically significant. In our study, echocardiographic, histological, and image-based analyses were performed in a blinded manner.

## 3. Results

### 3.1. Isolation and Characterization of Gq-EVs from Lycium barbarum Juice

**Figure 1:**
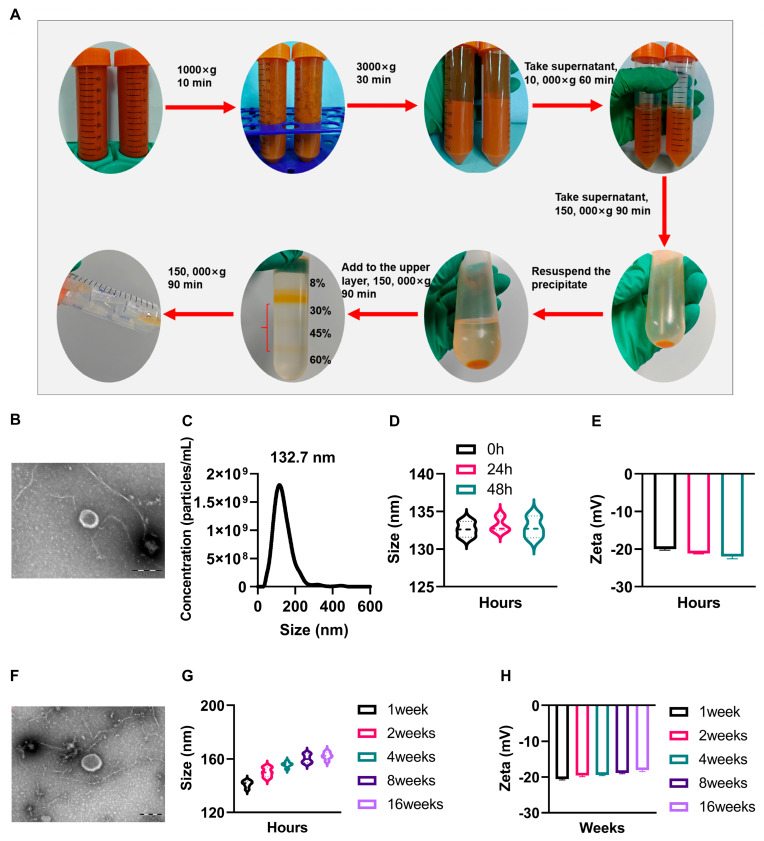
Extraction, preparation and characterization of Gq-EVs. (**A**) Extraction process of Gq-EVs. (**B**) Transmission electron microscopy image of Gq-EVs stored at 4 °C. (**C**) Average particle size of Gq-EVs measured by nanoparticle tracking analysis (NTA) stored at 4 °C. (**D**) The particle size and (**E**) zeta potential measured using the Malvern Laser Particle Sizer stored at 4 °C. (**F**) Transmission electron microscopy image of Gq-EVs stored at −80 °C. (**G**) The particle size and (**H**) zeta potential measured using the Malvern Laser Particle Sizer stored at −80 °C. Data were summarized as means ± SD. NTA, nanoparticle tracking analysis.

Gq-EVs were extracted from *Lycium barbarum* juice by ultracentrifugation and purified by density gradient centrifugation (Figure 1A). Transmission electron microscopy showed that Gq-EVs were spherical or discoidal EVs with uniform shape storage at 4 °C (Figure 1B). Nanoparticle tracking analysis (NTA) revealed that Gq-EVs exhibited a uniform particle size with an average of 132.7 nm (Figure 1C), no detectable impurity peaks, and a concentration of approximately 2.45 × 10^11^ particles/mL. To evaluate the stability of Gq-EVs, we measured the Malvern particle size and zeta potential of Gq-EVs immediately after extraction and preparation (0 h) and after storage at 4 °C for 24 h, 48 h. The results showed that the average particle sizes of Gq-EVs were 132.6 nm at 0 h, 133.2 nm after 24 h, and 132.9 nm after 48 h of storage at 4 °C (Figure 1C). The corresponding zeta potentials were −20 mV, −21.1 mV, and −21.6 mV at three time points (Figure 1D). The particle size and zeta potential were observed at different time points, indicating that Gq-EVs remained stable during storage at 4 °C for 48 h. Transmission electron microscopy showed that Gq-EVs were spherical or discoidal EVs with a uniform shape after storage at −80 °C (Figure 1F). The Malvern particle size and zeta potential of Gq-EVs were measured immediately after extraction and preparation, and after storage at −80 °C for 1 week, 2 weeks, 4 weeks, 8 weeks, and 16 weeks. The results showed that the average particle sizes of Gq-EVs were 141.3 nm, 150.1 nm, 155.7 nm, 160.3 nm, and 162.4 nm after storage at −80 °C (Figure 1G). The corresponding zeta potentials were −20.6 mV, −19.6 mV, −19.4 mV, −18.9 mV, and −18.1 mV at different time points (Figure 1H). Characterization analyses indicated that the extracted Gq-EVs exhibited a relatively stable structure, uniform size distribution, high purity, and short-term stability when stored at 4 °C and −80 °C.

### 3.2. Non-Targeted Metabolomic Analysis of Gq-EVs

**Figure 2:**
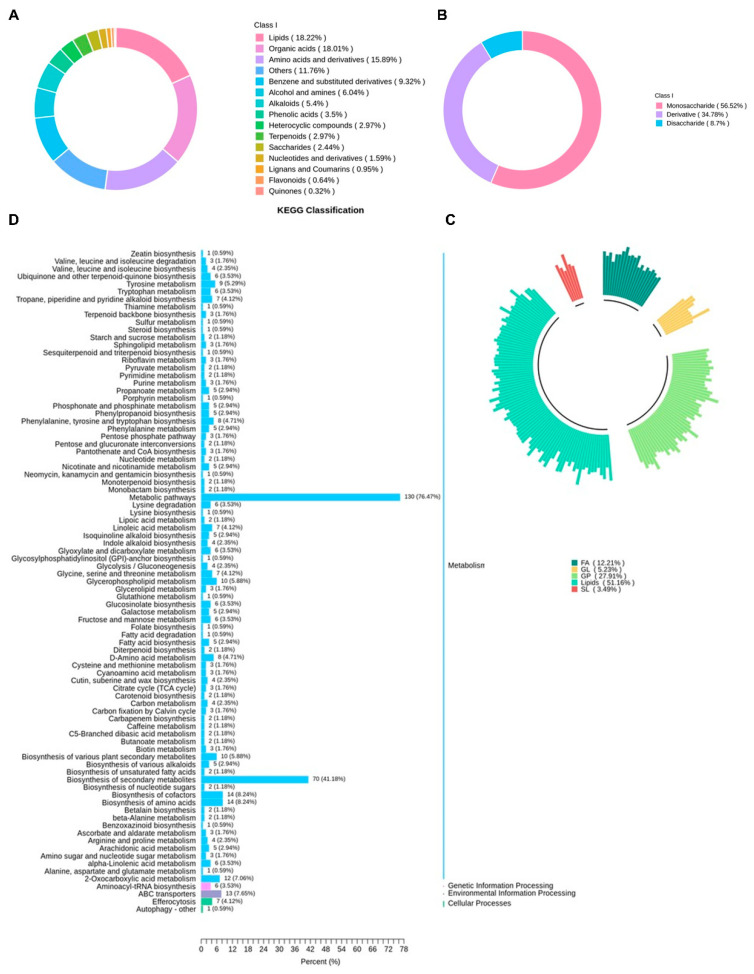
Metabolomic analysis for the component identification of Gq-EVs. (**A**) Components of Gq-EVs. (**B**) Carbohydrates in Gq-EVs. (**C**) Lipids in Gq-EVs. (**D**) KEGG pathways.

To explore the components of Gq-EVs, we conducted non-targeted metabolomic analysis on the Gq-EVs. We found that Gq-EVs contain lipids (18.22%), amino acids and their derivatives (15.89%), carbohydrates (2.44%), nucleotides and their derivatives (1.59%) (Figure 2A). Bioactive substances, such as alkaloids (5.4%), phenolic acids (3.5%), terpenoids (2.97%), lignins and coumarins (0.95%), flavonoids (0.64%) and quinones (0.32%), were also detected in Gq-EVs. Thirteen kinds of monosaccharides, such as glucose and L-fucose, and two kinds of disaccharides, such as sucrose, and eight carbohydrate derivatives constitute the carbohydrates in Gq-EVs (Figure 2B). Among the lipids in Gq-EVs, fat (51.16%) is the main component, followed by glycerophospholipid (27.91%). In addition, there were free fatty acids (12.21%), glycerolipid (5.23%), and sphingolipid (3.49%) (Figure 2C). KEGG pathway analysis revealed that Gq-EVs were enriched in metabolism-related pathways (Figure 2D).

### 3.3. Nebulized Inhalation of Gq-EVs Reduced Body Weight and Improved Fasting Glucose Levels in Mice with HFpEF

**Figure 3:**
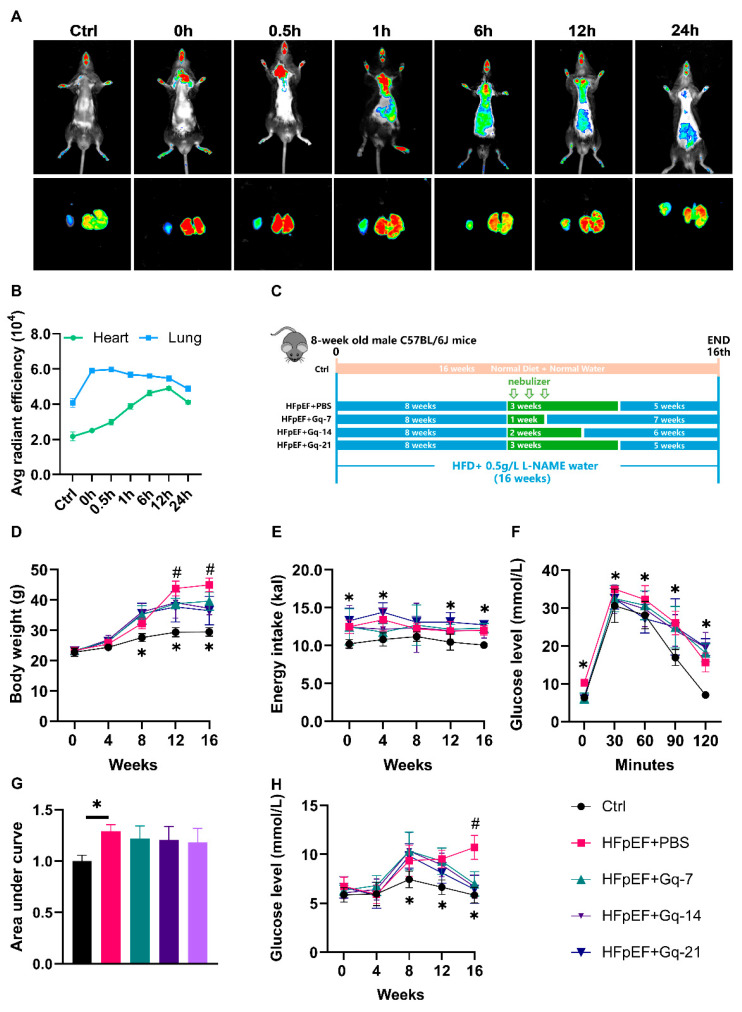
Effects of nebulized inhalation of Gq-EVs on mice with HFpEF. (**A**) In vivo imaging and ex vivo imaging of the heart and lungs. The color progression from blue to yellow and then to red indicates an increase in fluorescence intensity, reflecting a higher degree of Gq-EVs enrichment. (**B**) Statistics of the average fluorescence intensity of ex vivo imaging of heart and lung tissues. (**C**) Schematic diagram of the intervention of nebulized inhalation of Gq-EVs. (**D**) Body weight. (**E**) Energy intake. (**F**) GTT assay. (**G**) Area under the curve of GTT. (**H**) Fasting blood glucose levels. The data were summarized as means ± SD. *, *p* < 0.05 vs. Ctrl; #, *p* < 0.05 vs. HFpEF + PBS. GTT, glucose tolerance test; HFD, high-fat diet; L-NAME, N^ω^-nitro-L-arginine methyl ester. Abbreviations are the same as in Figure 1.

We used nebulized inhalation to administer Gq-EVs and evaluate their effects in mice with HFpEF. The distribution of Gq-EVs was tracked by in vivo small-animal imaging. The results showed that nebulized inhalation of DiR-labeled Gq-EVs could reach the thoracic cavity of mice and accumulate in the heart. In vitro imaging of the hearts and lungs of mice at different time points (0 h, 0.5 h, 1 h, 6 h, 12 h, and 24 h after the end of nebulization) revealed that DiR-labeled Gq-EVs first reached the lungs and transferred from the lungs to the heart over time. Significant accumulation in the heart was observed after 6 h, with the highest accumulation at 12 h, and a significant decrease after 24 h (Figure 3A,B). In addition, accumulation in the abdominal cavity began after 1 h, indicating that DiR-labeled Gq-EVs entered the systemic circulation. This suggests that nebulized inhalation of Gq-EVs can successfully reach and accumulate in the heart. Mice were induced with HFD combined with 0.5 g/L L-NAME in drinking water for 8 weeks (Figure S1), and then were given nebulized inhalation intervention of Gq-EVs for 1 week, 2 weeks, and 3 weeks, respectively, followed by continuous feeding for another 7 weeks, 6 weeks, and 5 weeks, respectively (Figure 3C). Compared with the Ctrl mice, the body weights of mice in each group with HFpEF increased, and there were significant differences after 8 weeks. Compared with the HFpEF + PBS group, the body weights of mice in the HFpEF + Gq-7, HFpEF + Gq-14, and HFpEF + Gq-21 groups decreased at the 12th and 16th weeks (Figure 3D). The energy intake of mice in each HFpEF group was more than that in the control group on the 4th, 8th, 12th, and 16th weeks (Figure 3E). Changes in body weight and energy intake indicate that Gq-EVs may exert a weight-reducing effect. Furthermore, statistical analysis of the weights of murine brown adipose tissue, subcutaneous adipose, and epididymal adipose demonstrated that Gq-EVs can increase brown adipose tissue while reducing subcutaneous and epididymal fat (Figure S2). The results of the glucose tolerance test (GTT) showed that mice in each HFpEF group all had abnormal glucose tolerance, and nebulized inhalation of Gq-EVs had no significant improvement effect on GTT (Figure 3F,G). Notably, nebulized inhalation of Gq-EVs improved the fasting blood glucose of mice, with a statistically significant difference observed at the 16th week (Figure 3H).

### 3.4. Nebulized Inhalation of Gq-EVs Improved Cardiac Function in Mice with HFpEF

**Figure 4:**
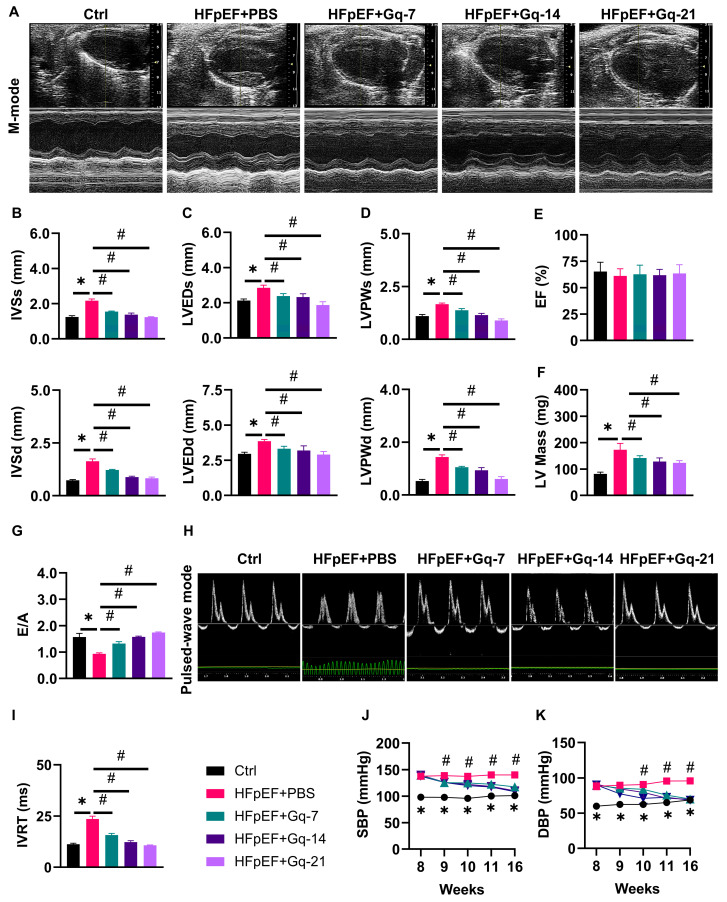
Nebulized inhalation of Gq-EVs improved cardiac function in mice with HFpEF. (**A**) B-mode and M-mode echocardiography. The yellow lines are the M-mode cursor. Left ventricular structural indicators (**B**) IVSS, IVST, (**C**) LVEDd, LVEDs, (**D**) LVPWd, LVPWs were determined. (**E**) EF%. (**F**) LV Mass. (**G**) E/A ratio of mitral valve orifice blood flow pattern. (**H**) Pulsed-mode echocardiography. The green lines are heart rate of mice. (**I**) IVRT. (**J**) SBP and (**K**) DBP were measured. The data were summarized as means ± SD. *, *p* < 0.05 vs. Ctrl; #, *p* < 0.05 vs. HFpEF + PBS. DBP, diastolic blood pressure; EF%, ejection fraction percentage; LVEDd, left ventricular end-diastolic diameter; LVEDs, left ventricular end-systolic diameter; LVPWd, left ventricular posterior wall thickness at end-diastole; LVPWs, left ventricular posterior wall thickness at end-systole; IVRT, Isovolumic relaxation time; IVSs, interventricular septum thickness at end-systolic; IVSd, interventricular septum thickness at end-diastolic; SBP, systolic blood pressure. Abbreviations are the same as in Figure 3.

To determine the improvement effect of nebulized inhalation of Gq-EVs on the cardiac function of HFpEF mice, echocardiography was performed to evaluate the structure and function of the left ventricle. Myocardial hypertrophy and diastolic dysfunction were observed in the HFpEF mouse group through B-mode and M-mode echocardiography on the 16th week (Figure 4A). Nebulized inhalation of Gq-EVs improved cardiac function in mice to varying degrees, with the most significant effect achieved after a 3-week nebulization intervention (Figure 4A). Analysis of the left ventricular structural indices on the 16th week, including the interventricular septum thickness at end-systole (IVSs), interventricular septum thickness at end-diastole (IVSd), left ventricular end-diastolic diameter (LVEDd), left ventricular end-systolic diameter (LVEDs), left ventricular posterior wall thickness at end-diastole (LVPWd), left ventricular posterior wall thickness at end-systole (LVPWs) revealed that nebulized inhalation of Gq-EVs for different durations improved the left ventricular structure and diastolic function in HFpEF mice (Figure 4B–D). Longitudinal echocardiography in M-mode showed that the ejection fraction (EF) remained unchanged in all groups (Figure 4E). Left ventricular mass (LV Mass), as well as diastolic function indices such as the isovolumic relaxation time (IVRT) and the E/A ratio of the mitral valve orifice blood flow, showed that nebulized inhalation of Gq-EVs for different durations reduced the mass of the left ventricle and improved diastolic function in HFpEF mice (Figure 4F–I). When monitoring the blood pressure of mice, we found that nebulized inhalation of Gq-EVs could reduce the systolic blood pressure (SBP) and diastolic blood pressure (DBP) in HFpEF mice, with significant statistical differences at the 9th, 10th, 11th, and 16th weeks (Figure 4J,K). In summary, these results indicate that nebulized inhalation of Gq-EVs alleviates left ventricular remodeling and diastolic dysfunction in HFpEF, with the most significant effect observed after a 3-week nebulization.

### 3.5. Nebulized Inhalation of Gq-EVs Alleviated Cardiac Pathological Damage in Mice with HFpEF

**Figure 5:**
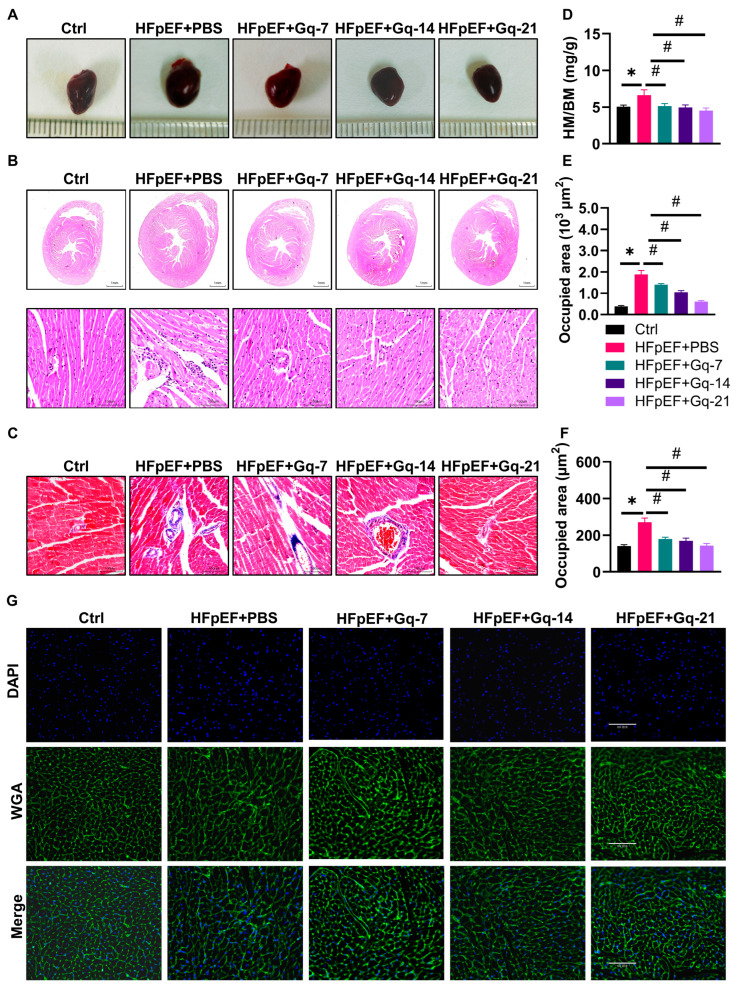
Nebulized inhalation of Gq-EVs alleviated cardiac pathological damage in mice with HFpEF. (**A**) Gross view of cardiac tissue. (**B**) H&E staining of cardiac tissue. (**C**) Masson’s trichrome staining was performed to assess (**E**) the deposition of mature fibers. (**D**) Cardiac organ index. (**F**) Statistics of (**G**) WGA staining in cardiac tissue. The data were summarized as means ± SD. *, *p* < 0.05 vs. Ctrl; #, *p* < 0.05 vs. HFpEF + PBS. Abbreviations are the same as in Figure 1.

Gross heart photographs and H&E staining showed that the hearts of mice in the HFpEF group were significantly hypertrophied, and nebulized inhalation of Gq-EVs effectively improved this condition, with the greatest effect observed in the HFpEF + Gq-21 group (Figure 5A,B). H&E staining of cardiac tissue revealed that the myocardial tissue of mice in the HFpEF group showed obvious disorder accompanied by infiltration of inflammatory cells. In the three groups receiving Gq-EVs nebulized inhalation, the disorder of myocardial tissue was reversed, and the infiltration of inflammatory cells was significantly reduced (Figure 5B). Nebulized inhalation of Gq-EVs reduced collagen fiber deposition in cardiac tissue, with the most pronounced improvement in fibrosis observed in the HFpEF + Gq-21 group (Figure 5C). Nebulized inhalation of Gq-EVs reversed cardiac organ indices in HFpEF hearts (Figure 5D). In addition, cardiomyocytes in the HFpEF + PBS group showed obvious hypertrophy and thickening of the cell membrane, which were effectively reversed by nebulized inhalation of Gq-EVs (Figure 5F,G). Nebulized inhalation of Gq-EVs improved cardiac pathological damage in HFpEF mice in multiple aspects.

### 3.6. The TP53-Related Signaling Pathway May Potentially or Partially Participate in the Therapeutic Effects of Gq-EVs on HFpEF

**Figure 6:**
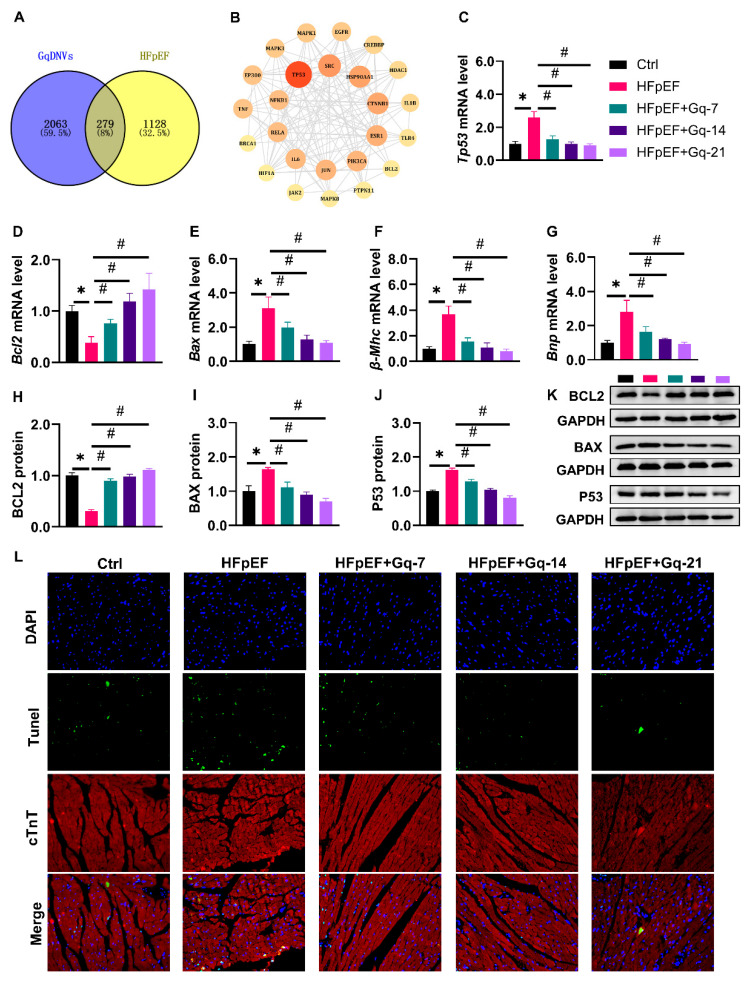
The *TP53*-related signaling pathway may potentially or partially participate in the therapeutic effects of Gq-EVs on HFpEF. (**A**) Venn diagram of targets between Gq-EVs and HFpEF. (**B**) Interaction network diagram of the top 25 targets. (**C**) *Tp53* mRNA levels. (**D**) *Bcl2* mRNA levels. (**E**) *Bax* mRNA levels. (**F**) *β-Mhc* mRNA levels. (**G**) *Bnp* mRNA levels. (**H**) Statistics of BCL2 protein levels. (**I**) Statistics of BAX protein levels. (**J**) Statistics of P53 protein levels. (**K**) Expression of BCL2, BAX, and P53 protein. (**L**) Co-staining of cardiomyocytes in cardiac tissue with TUNEL. The data were summarized as means ± SD. *, *p* < 0.05 vs. Ctrl; #, *p* < 0.05 vs. HFpEF + PBS. *Bnp*, brain natriuretic peptide; *β-Mhc*, β-myosin heavy chain. Abbreviations are the same as in Figure 1.

As extracellular vesicles contain various bioactive components, Gq-EVs may improve HFpEF by regulating multiple pathways and acting on multiple targets. Therefore, we investigated the molecular mechanism of the improvement effect of Gq-EVs on HFpEF. Using database analysis, we identified the regulatory targets of Gq-EVs and the disease-related targets of HFpEF patients, and determined their intersection, yielding 279 common targets (Figure 6A). A Protein–protein interaction (PPI) network was constructed, and topological parameters such as the node degree (i.e., the number of other targets interacting with the target) and betweenness centrality (which measures the importance of the target as a bridge connecting different modules in the network) of each target were calculated. The top 25 targets were selected for display based on the node degree and betweenness centrality. PPI network analysis identified TP53 with high node degree and betweenness centrality (Figure 6B). We found that enolase1, which has the highest abundance in proteins, and atamiR156c3p in miRNAs all exhibit direct binding to TP53 (Figure S3). The total docking scores were −264.38 and −333.24, indicating that ata-miR156c-3p and Enolase1 exhibit extremely strong binding affinity to TP53 with highly stable conformations (values ≤−200 signify strongly stable binding, corresponding to a naturally credible binding pattern). The Confidence Scores of 0.9078 and 0.9750 suggest that the docked conformations are highly likely to approximate the native structure (range: 0~1; ≥0.7 indicates highly reliable binding with exceptional model credibility). Furthermore, *Lycium ruthenicum* Murray-derived exosome-like nanovesicles can inhibit Aβ-induced apoptosis in PC12 cells through the MAPK and PI3K/AKT signaling pathways, with miRNAs playing a pivotal role [22]. Moreover, atamiR156c3p can inhibit apoptosis. Another study showed that loss of myocardial *TP53* delays HFpEF onset in a high-fat diet and L-NAME-challenged murine model [40]. TP53 is closely related to cardiomyocyte apoptosis [41,42]. So the *TP53*-related signaling pathway may potentially or partially participate in the therapeutic effects of Gq-EVs on HFpEF. Further verification of *Tp53* mRNA levels in mice (Figure 6C) showed a significant increase in *Tp53* expression in the cardiac tissue of HFpEF mice, which was markedly reduced following Gq-EVs intervention. HFpEF resulted in an enhanced expression of pro-apoptotic factor Bcl-2-associated X protein (*Bax*) mRNA, β-myosin heavy chain (*β-Mhc*) and brain natriuretic peptide (*Bnp*) mRNA, and reduced expression of anti-apoptotic factor B-cell lymphoma 2 (*Bcl2*) mRNA (Figure 6D–G). Enhanced expression of BAX protein and P53 protein, and reduced expression of BCL2 protein were determined in the cardiac tissue of HFpEF mice (Figure 6H–K). These effects were abolished by Gq-EVs (Figure 6C–K). Through co-staining and quantitative analysis of cardiomyocytes with TUNEL, we observed that the cardiomyocytes in the HFpEF group were arranged disorderly and apoptosis was obvious, and inhalation of Gq-EVs had a rescue effect on cardiomyocyte apoptosis (Figure 6L, Figure S4).

### 3.7. Gq-EVs Improved Cardiomyocyte Apoptosis and Regulated the TP53 Pathway

**Figure 7:**
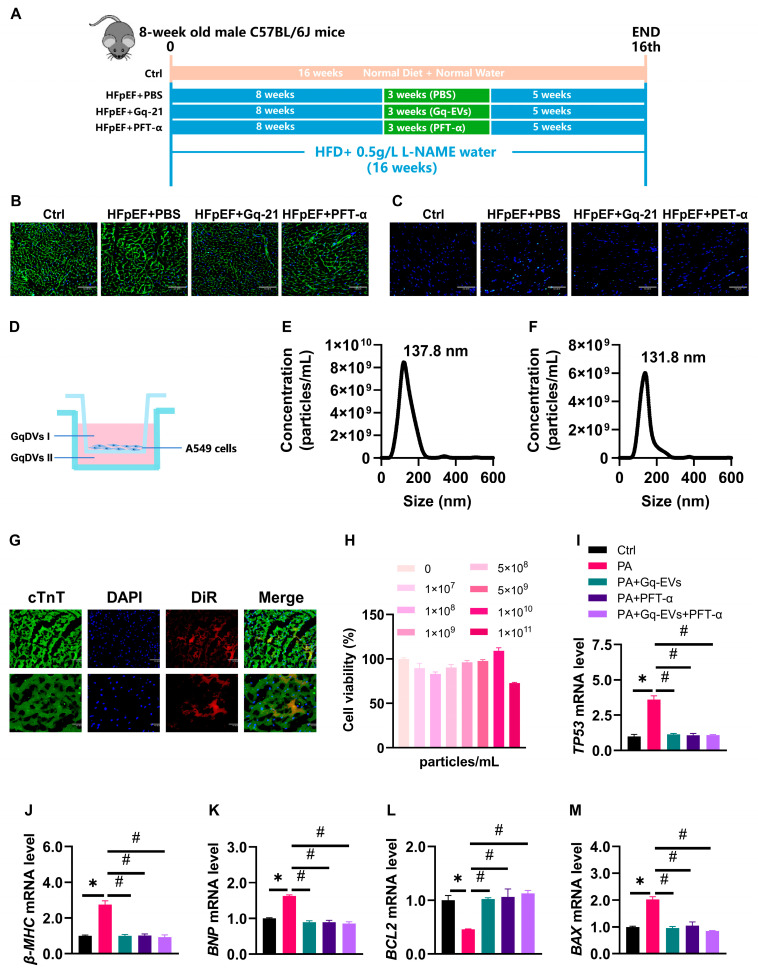
Gq-EVs improved cardiomyocyte apoptosis and regulated the *TP53* pathway. (**A**) Schematic diagram of the intervention of Gq-EVs and PFT-α in mice. (**B**) WGA staining and (**C**) TUNEL staining of cardiac tissue. (**D**) Trans-pulmonary cell experiment of Gq-EVs. (**E**) Average particle size of Gq-EVs measured by NTA in the upper chamber of the transwell chamber. (**F**) Average particle size of Gq-EVs measured by NTA in the lower chamber. (**G**) Co-staining of Gq-EVs and cTnT in cardiomyocytes. (**H**) CCK-8 assay of cells treated with Gq-EVs. (**I**) *TP53* mRNA levels. (**J**) *β-MHC* mRNA levels. (**K**) *BNP* mRNA levels. (**L**) *BCL2* mRNA levels. (**M**) *BAX* mRNA levels. The data were summarized as means ± SD. *, *p* < 0.05 vs. Ctrl; #, *p* < 0.05 vs. PA. *BAX*, Bcl-2-associated X protein; *BCL2*, B-cell lymphoma 2; *BNP*, brain natriuretic peptide; *β-MHC*, β-myosin heavy chain; cTnT, cardiac troponin T; PFT-α, Pifithrin-α. Abbreviations are the same as in Figure 1.

Subsequently, we examined *TP53*-related apoptotic signaling in vivo to explore its potential involvement in myocardial hypertrophy and cardiomyocyte apoptosis in HFpEF (Figure 7A). WGA staining showed that the *TP53* inhibitor Pifithrin-α (PFT-α) could alleviate myocardial cell hypertrophy, and nebulized inhalation of Gq-EVs for 3 weeks reversed myocardial hypertrophy better in HFpEF mice (Figure 7B). In addition, after PFT-α or Gq-EVs intervention, cardiac tissue cell apoptosis was significantly reduced (Figure 7C). Transcellular experiments showed that Gq-EVs traversed the pulmonary barrier in vitro, and live-imaging analyses demonstrated their partial uptake by cardiomyocytes in mice. These data indicate that small Gq-EVs can pass through lung tissue and be taken up by cardiomyocytes partially (Figure 7D–G). In the CCK-8 experiment, we determined that the appropriate dose of Gq-EVs for intervening in human cardiomyocyte AC16 was 1 × 10^10^ particles/mL (Figure 7H). We used the *TP53*-specific inhibitor, PFT-α, to suppress the activity of TP53 at the transcriptional level, thereby blocking the transcriptional activation of TP53 downstream target genes. Gq-EVs can reduce the mRNA level of *TP53* and downregulate the mRNA levels of the myocardial hypertrophy markers *β-MHC* and *BNP* (Figure 7I–K). Its effect may be related to upregulating *BCL2* and downregulating *BAX* mRNA (Figure 7L,M), similar with the *TP53* inhibitor PFT-α. This suggests that Gq-EVs can alleviate the toxicity of 500 μM palmitic acid (PA) to AC16 cells and reduce cardiomyocyte apoptosis, which may indicate potential or partial involvement of *TP53*-related signaling.

### 3.8. Gq-EVs Alleviated the PA-Induced Cardiomyocyte Apoptosis and Were Potentially or Partially Associated with Alterations in the TP53 Mitochondrial Pathway

**Figure 8:**
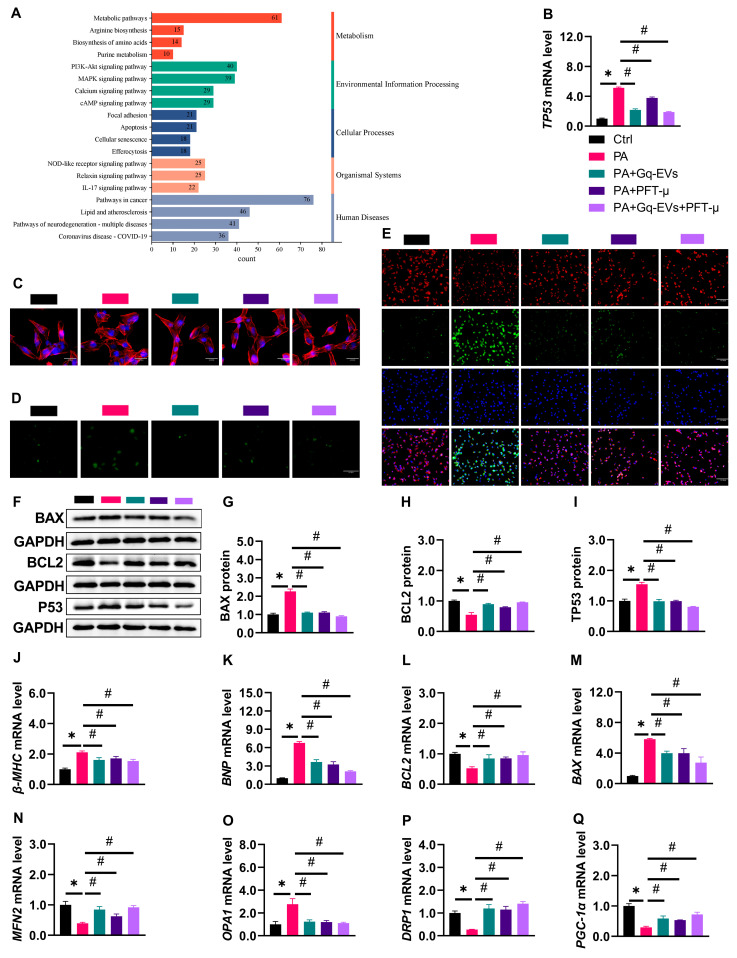
Gq-EVs alleviated the PA-induced cardiomyocyte apoptosis and were potentially or partially associated with alterations in the *TP53* mitochondrial pathway. (**A**) KEGG pathway map related to the enrichment of Gq-EVs and HFpEF. (**B**) *TP53* mRNA levels. (**C**) Phalloidin staining. (**D**) TUNEL staining. (**E**) JC-1 staining of cardiomyocytes. (**F**) Expression of BAX, BCL2, and P53 proteins. (**G**) Statistics of BAX protein levels. (**H**) Statistics of BCL2 protein levels. (**I**) Statistics of P53 protein levels. (**J**) *β-MHC* mRNA levels. (**K**) *BNP* mRNA levels. (**L**) *BCL2* mRNA levels. (**M**) *BAX* mRNA levels. (**N**) *MFN2* mRNA levels. (**O**) *OPA1* mRNA levels. (**P**) *DRP1* mRNA levels. (**Q**) *PGC-1α* mRNA levels. The data were summarized as means ± SD. *, *p* < 0.05 vs. Ctrl; #, *p* < 0.05 vs. PA. *DRP1*, Dynamin-related protein 1; *OPA1*, Optic atrophy 1; PFT-μ, Pifithrin-μ; *PGC-1α*, Peroxisome proliferator-activated receptor gamma coactivator 1-alpha; *MFN2*, Mitofusin 2. Abbreviations are the same as in Figure 1.

*TP53* can increase the mitochondrial membrane permeability by upregulating the expression of pro-apoptotic genes (such as *Bax*), leading to the release of cytochrome C and the activation of the caspase cascade reaction, ultimately resulting in cell apoptosis [43,44]. We found that Gq-EVs are rich in carbohydrates and lipids, and these components were predicted and associated with metabolic processes through non-targeted metabolomics analysis. In addition, Gq-EVs are associated with the metabolic pathways enriched in HFpEF (Figure 8A). We want to further explore whether the regulation of cardiomyocyte apoptosis by Gq-EVs is potentially or partially associated with the *TP53* mitochondrial pathway. PFT-μ, a specific inhibitor of *TP53* mitochondrial function, can prevent the translocation of *TP53* to the mitochondria, thereby inhibiting the *TP53*-dependent mitochondrial apoptotic pathway [45,46]. Gq-EVs, PFT-μ, and PFT-μ + Gq-EVs downregulated the mRNA levels of *TP53* mRNA level (Figure 8B), and alleviated PA-induced cardiomyocyte hypertrophy (Figure 8C) and cardiomyocyte apoptosis (Figure 8D). The decrease in mitochondrial membrane potential is a hallmark event in the early stage of cell apoptosis. The decline of the mitochondrial membrane potential and the occurrence of cell apoptosis can be detected by the transition of JC-1 (Figure 8E). Gq-EVs downregulated the expression of BAX and P53 protein, both of which were increased under PA, and reactivated the expression of BCL2 protein (Figure 8F–I). Further investigation showed that Gq-EVs reduced the expression of the PA-induced expression of *β-MHC*, *BNP*, *BAX*, and *OPA1* mRNA, and reversed the PA-prohibited expression of *BCL2*, *MFN2*, *DRP1*, and *PGC-1α* as markers for apoptosis and mitochondrial dysfunction (Figure 8J–Q). The effect of Gq-EVs, PFT-μ, and PFT-μ + Gq-EVs is similar. This indicated that Gq-EVs are potentially or partially associated with reduced cardiomyocyte apoptosis, with possible involvement of the mitochondrial *TP53* pathway.

### 3.9. Schematic Diagram Illustrating the Proposed Actions of Gq-EVs in HFpEF

**Figure 9:**
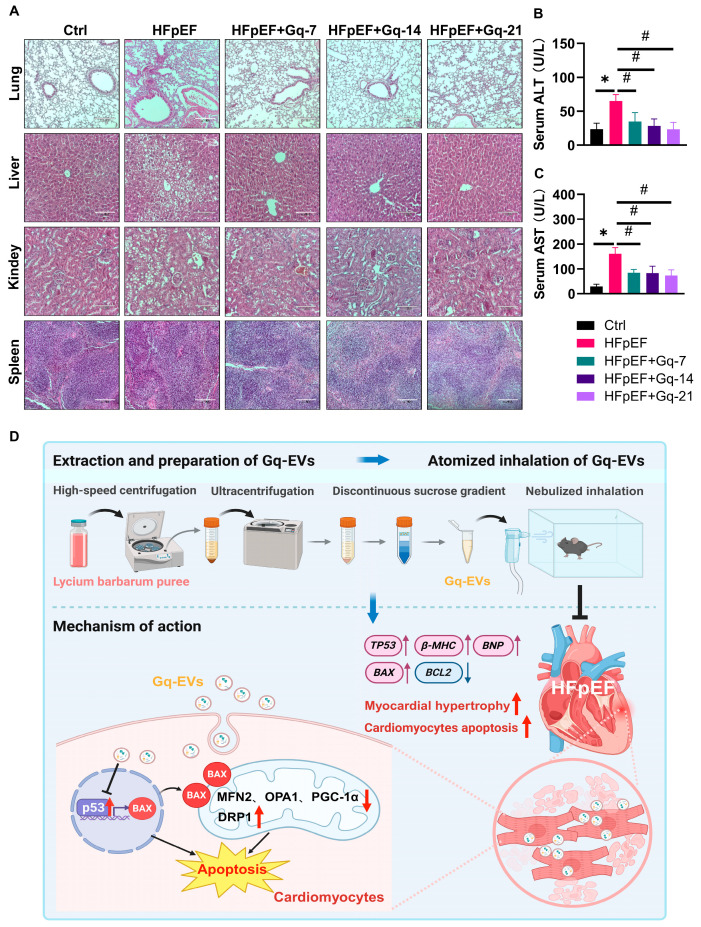
Schematic diagram illustrating the proposed actions of Gq-EVs in HFpEF. (**A**) H&E staining of lung, spleen, liver, and kidney tissues. (**B**) Serum ALT level. (**C**) Serum AST level. (**D**) Mechanism diagram for the actions of Gq-EVs on HFpEF. Nebulized inhalation of Gq-EVs allows their delivery to cardiac tissue. In HFpEF, cardiomyocyte apoptosis and myocardial hypertrophy are associated with increased *TP53* expression, activation of mitochondrial apoptotic signaling (e.g., BAX), and upregulation of heart failure markers (β-MHC and BNP), accompanied by reduced anti-apoptotic signaling (BCL2). Gq-EVs treatment is associated with attenuation of cardiomyocyte apoptosis and improvement of cardiac hypertrophy, potentially involving modulation of *TP53*-associated mitochondrial pathways and mitochondrial dynamics–related proteins. The data were summarized as means ± SD. *, *p* < 0.05 vs. Ctrl; #, *p* < 0.05 vs. ALT, alanine aminotransferase; AST, Aspartate Aminotransferase. Abbreviations are the same as in Figure 1.

It is crucial to evaluate the potential side effects of Gq-EVs on organs other than the heart. Pathological analysis showed that the tissue structures of organs such as the lungs, spleen, liver, and kidneys of the treated mice were similar to those in healthy mice, and no organ toxicity or inflammation was observed (Figure 9A). In addition, blood tests indicated that nebulized inhalation of Gq-EVs had no significant impact on the liver function of the mice (Figure 9B,C). Nebulized inhalation of Gq-EVs enables their delivery to the heart, where they are potentially or partially associated with reduced TP53 expression and modulation of TP53-related mitochondrial apoptotic signaling, leading to attenuation of cardiomyocyte apoptosis and improvement of cardiac hypertrophy in HFpEF mice (Figure 9D).

## 4. Discussion

Through this study, we found that in mice with HFpEF induced by HFD combined with L-NAME, cardiac function was impaired and diastolic dysfunction occurred. Inhaled Gq-EVs repaired the damaged cardiac tissue and improved the cardiac structure and function in HFpEF mice. The beneficial effects of Gq-EVs on cardiac function in HFpEF mice may be potentially or partially associated with reduced cardiomyocyte apoptosis and involvement of the *TP53* mitochondrial pathway. Gq-EVs, carrier-free systems, are easy to obtain and have high drug-loading capacity, making them the preferred choice in the pharmaceutical industry. Gq-EVs, a mixture derived from the plant wolfberry, contain a variety of natural bioactive substances with excellent biocompatibility, anti-inflammatory, and antioxidant properties, which can enhance therapeutic effects when co-delivered with drugs.

At present, the treatment strategy for HFpEF includes SGLT2 drug therapy, MRA, ARNIS, GLP-1 receptor agonists, combined with management of comorbidity and lifestyle interventions [6,47,48,49]. These traditional drug therapies have certain side effects, which can increase the risk of urinary tract infections, cause hypoglycemia, hypokalemia, hyponatremia, dehydration, hypotension, affect renal function, and be accompanied by gastrointestinal reactions such as nausea, vomiting, and diarrhea [8,50,51,52,53,54]. Therefore, there is a need to explore safe, non-toxic, low-cost natural compounds with minimal side effects to improve outcomes in HFpEF. In this study, we isolated Gq-EVs from *Lycium barbarum* juice. *Lycium barbarum* is a substance that can be used both as food and medicine, and its constituents have been generally recognized as safe and non-toxic. As an ultra-centrifuged extract of *Lycium barbarum*, Gq-EVs have a simple preparation process, making them highly feasible for clinical application. Meanwhile, we found that Gq-EVs contain components like those of *Lycium barbarum* [55]. As a mixture, Gq-EVs are rich in various anti-inflammatory and antioxidant substances, such as amino acids and their derivatives, carbohydrates, alkaloids, phenolic acids, terpenoids, lignins and coumarins, flavonoids and quinones, which can exert additive or synergistic effects. The extraction of Gq-EVs from *Lycium barbarum* juice not only provides a potential intervention strategy for HFpEF but also provides new ideas for the sustainable development, transformation, and utilization of *Lycium barbarum* and its product resources.

Previous EV delivery methods for treating heart diseases are invasive and limited by large surgical incisions, low efficiency, and non-reproducibility [56,57,58]. Our study verified the potential of inhaled Gq-EVs to cross the blood-gas barrier and localize to the damaged heart. Repeated administration can promote the enrichment of Gq-EVs in cardiac tissue, providing sufficient Gq-EVs for the damaged heart. Inhalation administration is a non-invasive treatment method, which has been proven to be feasible in treating many diseases, not just respiratory diseases [59,60,61]. Administration via the tail-vein injection directly enters the systemic circulation and then is distributed throughout the body through the pumping function of the heart, which can ensure that EVs quickly reach the target site, but the intervention is invasive, with significantly higher hepatorenal toxicity and immune hyperactivation, and requires certain technical support [62,63]. When administered orally, EVs need to be absorbed through the gastrointestinal tract and may be affected by gastric acid and digestive enzymes. Subcutaneous and intramuscular injections directly inject exosomes into local tissues, which are then gradually absorbed into the systemic circulation with limited effects. Compared with other administration methods, nebulized inhalation can fully exert the drug intervention effect and is non-invasive, safe, and low in immunogenicity. The biodistribution experiments show the presence of labeled vesicles in the cardiac region after inhalation, and quantitative evidence of efficient myocardial delivery is necessary for future research.

Gq-EVs contain a variety of bioactive components, which may improve HFpEF by regulating multiple pathways and acting on multiple targets. In our study, histopathological analyses using H&E, Masson’s trichrome, and WGA staining showed that Gq-EVs treatment markedly improved inflammatory cell infiltration, myocardial tissue disorder, cardiac fibrosis, and myocardial hypertrophy in HFpEF mice. The improvement effect was enhanced as the intervention time was prolonged. Although we subsequently combined bioinformatics technology with network pharmacology to focus on key targets, the multi-target and multi-pathway regulatory effects of Gq-EVs should not be overlooked. This study employed a network pharmacology approach to predict the potential targets of Gq-EVs and identified *TP53*-related signaling as a possible molecular target. Although this analysis provides valuable hypotheses for exploring the mechanism of action of Gq-EVs, as network pharmacology technology only qualitatively predicts drug components and targets, and clear pharmacological effects need to be verified through animal experiments or even clinical trials, further biological experimental validation is still required. Therefore, the network pharmacology-based analysis in this paper should be regarded as preliminary clues for mechanistic investigation. Future studies need to combine in vivo and in vitro experiments to functionally validate *TP53* and related pathways and more accurately elucidate the mechanisms of Gq-EVs in HFpEF.

Based on the structural and particle-size characteristics of exosomes, we chose nebulized inhalation as the method to intervene in HFpEF mice. Nebulized inhalation administration is a more direct approach. It could avoid the losses during the digestion, absorption, and metabolism processes of oral administration and the invasive limitations of intravenous injection intervention, thus fully exerting the effect of drug intervention. There are significant differences between nebulized inhalation and oral gavage in terms of cardioprotective effects, bioavailability, and systemic toxicity. The initial process following inhalation is drug deposition; particles smaller than 0.5 micrometers do not deposit in the lungs but typically diffuse into the bloodstream and reach other target organs [64]. Comparison of the distribution patterns of turmerone after oral administration versus inhalation revealed significantly higher relative concentrations (turmerone levels in each organ/serum) in the lungs, brain, heart, and kidneys in the inhalation group compared to the oral administration group [65]. The inhalation administration of sinapine thiocyanate demonstrated a 2.7-fold increase in AUC compared with oral gavage, with a corresponding enhancement in systemic exposure and a prolonged systemic residence time than tail vein injection [66]. Inhalation delivery of nanoparticles is an effective, safe, and noninvasive strategy for targeted treatment of heart diseases [60,61,67,68]. Mice that inhaled stem cell-derived exosomes for 10 min daily over a period of 7 consecutive days exhibited significant improvement in left ventricular function. Histological studies further confirmed that this therapy reduced fibrotic tissue and inhibited myocardial cell apoptosis. Additionally, safety evaluations demonstrated no toxicity to multiple organs including the lungs, spleen, liver, and kidneys [28]. We are also strongly interested in the differences among nebulized inhalation, oral gavage, and intravenous injection interventions, so our future studies could explore the differences in terms of cardioprotective effects, bioavailability, and systemic toxicity.

Gq-EVs intervention significantly reduced body weight in mice without altering their average daily energy intake in our study. This suggests that the intervention may increase the mice’s total energy expenditure. Consistent with behavioral observations during the experiment, mice in the Gq-EVs intervention group exhibited significantly higher levels of spontaneous activity (such as more frequent running and exploratory behavior). Furthermore, statistical analysis of the weights of murine brown adipose tissue, subcutaneous fat, and epididymal fat demonstrated that Gq-EVs can increase brown adipose tissue while reducing subcutaneous and epididymal fat (white adipose tissue). Combined with behavioral observations (more active mice in the Gq-EVs intervention group) and adipose tissue analyses, we conclude that this phenomenon suggests Gq-EVs may significantly enhance systemic energy expenditure in mice by reducing white adipose tissue (particularly subcutaneous and epididymal fat) and activating classical brown adipose tissue, ultimately leading to weight reduction. To investigate the effects of Gq-EVs on energy metabolism in HFpEF mice, future research will directly measure mice’s oxygen consumption using metabolic cages to quantify their total energy expenditure and respiratory quotient, as well as additional research on how exactly brown adipose tissue activation and white adipose tissue reduction occur, thereby exploring the precise metabolic mechanisms underlying improved cardiac function and exercise tolerance in HFpEF mice.

In our study, Gq-EVs significantly reduced both systolic and diastolic blood pressure, body weight, and fasting glucose levels. Since improvements in these systemic hemodynamic and metabolic parameters are themselves recognized as key modulators of myocardial remodeling, fibrosis, and diastolic function in HFD/L-NAME-induced HFpEF, we cannot fully distinguish whether the observed cardioprotective effects are primarily due to direct cardiomyocyte-targeted actions of Gq-EVs (potentially via *TP53*-associated mechanisms) or are largely secondary to these systemic improvements. Nevertheless, the robust *TP53* transcriptional signature detected in cardiomyocytes following Gq-EVs uptake, together with in vitro evidence of TP53-mediated anti-fibrotic signaling, supports a direct component. However, similar studies have reported that metformin improves myocardial metabolic abnormalities in SHR, while simultaneously preventing systolic dysfunction and left ventricular hypertrophy [69]. Future studies employing controlled blood pressure lowering, weight matching, or normalized glucose levels in parallel with Gq-EVs treatment, as well as conditional *TP53* deletion experiments, will be essential to definitively disentangle these interconnected physiological axes and quantify the proportion of benefit attributable to direct cardiac *TP53* activation versus indirect systemic modulation. Although the above results are promising, this study has several limitations. We investigated and validated the effects of Gq-EVs on myocardial hypertrophy in HFpEF mice and in AC16 cells. However, further clinical studies are necessary to confirm their efficacy and safety in humans. In addition, delivery via inhalation may result in limited cardiac retention of exosomes, requiring continuous administration to achieve sufficient cardiac enrichment of Gq-EVs. To improve cardiac targeting, future studies could explore strategies to modify Gq-EVs, such as conjugation with a cardiac-homing peptide to enhance accumulation in injured myocardium [70,71] or the development of controlled-release systems to enable sustained delivery [72,73]. In our study, mice were induced with HFD combined with 0.5 g/L L-NAME in drinking water to establish HFpEF [74]. Due to the heterogeneity of HFpEF, this animal model mainly represents a metabolic-related HFpEF phenotype, which is a limitation of the study. The model used (HFD + L-NAME) largely simulates the metabolic phenotype of HFpEF. Since HFpEF is a heterogeneous disease in humans, future research will refine by testing in other models representing different causes and disease phenotypes to confirm the broader applicability of the therapy. In addition, repeated inhalation of plant-derived extracellular vesicles may potentially induce pulmonary or immune responses. We will explore pulmonary inflammatory markers, bronchoalveolar lavage fluid, eosinophilic infiltration, or allergic reactions in future studies.

## 5. Conclusions

In this study, we demonstrated that Gq-EVs with high biological activity and at a relatively high concentration can be efficiently obtained from *Lycium barbarum* juice. Nebulized inhalation of Gq-EVs for 7, 14, and 21 days significantly improved the diastolic dysfunction in mice with HFpEF induced by an HFD combined with 0.5 g/L L-NAME in drinking water. Gq-EVs treatment attenuated excessive myocardial hypertrophy, inflammatory cell infiltration, and collagen deposition in the heart tissue. In addition, both in vivo and in vitro experiments showed that Gq-EVs could reach the injured cardiac tissue, improve mitochondrial function, and were associated with reduced cardiomyocyte apoptosis and amelioration of myocardial hypertrophy, potentially or partially involving *TP53*-related apoptotic signaling. Collectively, these findings identify inhalable Gq-EVs as a novel, non-invasive nanotherapeutic strategy that confers significant cardioprotective effects in a murine model of HFpEF.

## Data Availability

The original contributions presented in this study are included in the article/Supplementary Materials. Further inquiries can be directed to the corresponding authors.

## Supplementary Materials

The following supporting information can be downloaded at: https://www.mdpi.com/article/10.3390/nu18152564/s1. Figure S1: Serum BNP after 8 weeks of induction; Figure S2: Statistical analysis of the weights of murine brown adipose, subcutaneous adipose and epididymal adipose; Figure S3: Evidence for TP53 as a core target; Figure S4: Quantitative analysis for TUNEL staining; Table S1: Primer sequences.

## References

[1] Khan M.S., Shahid I., Bennis A., Rakisheva A., Metra M., Butler J. (2024). Global epidemiology of heart failure. Nat. Rev. Cardiol..

[2] Shi Y., Zhao L., Wang J., Liu X., Bai Y., Cong H., Li X. (2024). Empagliflozin protects against heart failure with preserved ejection fraction partly by inhibiting the senescence-associated STAT1-STING axis. Cardiovasc. Diabetol..

[3] Hamo C.E., DeJong C., Hartshorne-Evans N., Lund L.H., Shah S.J., Solomon S., Lam C.S. (2024). Heart failure with preserved ejection fraction. Nat. Rev. Dis. Primers.

[4] Liu L., Wang R., Strocchi S., Eroglu T., Nambiar N., Contreras S.V.L., Diezel S.A., Schiattarella G.G. (2025). Serum amyloid A in HFpEF and cardiometabolic diseases. Basic Res. Cardiol..

[5] Redfield M.M., Borlaug B.A. (2023). Heart Failure with Preserved Ejection Fraction: A Review. JAMA.

[6] Pandey A.K., Bhatt D.L., Pandey A., Marx N., Cosentino F., Pandey A., Verma S. (2023). Mechanisms of benefits of sodium-glucose cotransporter 2 inhibitors in heart failure with preserved ejection fraction. Eur. Heart J..

[7] Omote K., Verbrugge F.H., Borlaug B.A. (2022). Heart Failure with Preserved Ejection Fraction: Mechanisms and Treatment Strategies. Annu. Rev. Med..

[8] Kittleson M.M., Panjrath G.S., Bates K., Breathett K.K., Dixon D.L., Januzzi J.L., Mohammed S.F. (2026). Management of Heart Failure with Preserved Ejection Fraction: 2026 ACC Expert Consensus Decision Pathway: A Report of the American College of Cardiology Solution Set Oversight Committee. J. Am. Coll. Cardiol..

[9] Halimi S., Vergès B. (2014). Adverse effects and safety of SGLT-2 inhibitors. Diabetes Metab..

[10] Avogaro A., Delgado E., Lingvay I. (2018). When metformin is not enough: Pros and cons of SGLT2 and DPP-4 inhibitors as a second line therapy. Diabetes/Metab. Res. Rev..

[11] Chang J., Ambrosy A.P., Vardeny O., Van Spall H.G.C., Mentz R.J., Sauer A.J. (2024). Mineralocorticoid Antagonism in Heart Failure: Established and Emerging Therapeutic Role. JACC Heart Fail..

[12] Cacioli G., Gallone G., Verde A., Ciabatti M., Pidello S., Colombo V., De Fazio L., Peano V., Angeli G., De Donno F. (2025). Mechanisms and Prognosis of Intolerance to Angiotensin Receptor-Neprilysin Inhibitors in Advanced Heart Failure: Insights from Vasodilator Challenge. Can. J. Cardiol..

[13] Xu Z., Xu Y., Zhang K., Liu Y., Liang Q., Thakur A., Liu W., Yan Y. (2023). Plant-derived extracellular vesicles (PDEVs) in nanomedicine for human disease and therapeutic modalities. J. Nanobiotechnology.

[14] Feng J., Xiu Q., Huang Y., Troyer Z., Li B., Zheng L. (2023). Plant-Derived Vesicle-Like Nanoparticles as Promising Biotherapeutic Tools: Present and Future. Adv. Mater..

[15] Hao S., Yang H., Hu J., Luo L., Yuan Y., Liu L. (2024). Bioactive compounds and biological functions of medicinal plant-derived extracellular vesicles. Pharmacol. Res..

[16] Zhao B., Lin H., Jiang X., Li W., Gao Y., Li M., Yu Y., Chen N., Gao J. (2024). Exosome-like nanoparticles derived from fruits, vegetables, and herbs: Innovative strategies of therapeutic and drug delivery. Theranostics.

[17] Jin E., Yang Y., Cong S., Chen D., Chen R., Zhang J., Hu Y., Chen W. (2025). Lemon-derived nanoparticle-functionalized hydrogels regulate macrophage reprogramming to promote diabetic wound healing. J. Nanobiotechnology.

[18] Yi Q., Xu Z., Thakur A., Zhang K., Liang Q., Liu Y., Yan Y. (2023). Current understanding of plant-derived exosome-like nanoparticles in regulating the inflammatory response and immune system microenvironment. Pharmacol. Res..

[19] Tuo R., Huang Y., Chen X., Zhang Z., Li Z., Wang R., Dong R., Liu S., Gao D., Gu Q. (2025). Tangerine Peel Exosome-like Nanoparticles (TPELNs) Mitigate Intestinal Inflammation via Gut Microbiome Remodeling and Immune Modulation. J. Agric. Food Chem..

[20] Zhao W.J., Bian Y.P., Wang Q.H., Yin F., Yin L., Zhang Y.-L., Liu J.-H. (2022). Blueberry-derived exosomes-like nanoparticles ameliorate nonalcoholic fatty liver disease by attenuating mitochondrial oxidative stress. Acta Pharmacol. Sin..

[21] Shen H., Zhang M., Liu D., Liang X., Chang Y., Hu X., Gao W. (2025). Solanum lycopersicum derived exosome-like nanovesicles alleviate restenosis after vascular injury through the Keap1/Nrf2 pathway. Food Funct..

[22] Zhang Y., Zhang X., Kai T., Zhang L., Li A. (2024). Lycium ruthenicum Murray derived exosome-like nanovesicles inhibit Aβ-induced apoptosis in PC12 cells via MAPK and PI3K/AKT signaling pathways. Int. J. Biol. Macromol..

[23] You J.Y., Kang S.J., Rhee W.J. (2021). Isolation of cabbage exosome-like nanovesicles and investigation of their biological activities in human cells. Bioact. Mater..

[24] Yang S., Guo J., Chen D., Sun Z., Pu L., Sun G., Yang M., Peng Y. (2025). The Cardioprotective Effect of Ginseng Derived Exosomes via Inhibition of Oxidative Stress and Apoptosis. ACS Appl. Bio Mater..

[25] Ye C., Yan C., Bian S.J., Li X.-R., Li Y., Wang K.-X., Zhu Y.-H., Wang L., Wang Y.-C., Wang Y.-Y. (2024). Momordica charantia L.-derived exosome-like nanovesicles stabilize p62 expression to ameliorate doxorubicin cardiotoxicity. J. Nanobiotechnology.

[26] Liu Y., Qi H., Zong J., Li M., Yang Y., Li X., Li T., Cho J.Y., Yu T. (2024). Oral Piwi-Interacting RNA Delivery Mediated by Green Tea-Derived Exosome-Like Nanovesicles for the Treatment of Aortic Dissection. Adv. Healthc. Mater..

[27] Chen W., Zhao Y., Zhao Q., Zhou Y., Ma C., Dong L., Luo Y., Zhang Z., Chen F., Hu X. (2026). Lachnospiraceae-Derived Extracellular Vesicles Mediate the Cardioprotective Effects of Barley Leaf in Myocardial Infarction by Improving Intestinal Stem Cell Function. J. Extracell. Vesicles.

[28] Li J., Sun S., Zhu D., Mei X., Lyu Y., Huang K., Li Y., Liu S., Wang Z., Hu S. (2024). Inhalable Stem Cell Exosomes Promote Heart Repair After Myocardial Infarction. Circulation.

[29] Li M., Huang H., Wei X., Li H., Li J., Xie B., Yang Y., Fang X., Wang L., Zhang X. (2025). Correction: Clinical investigation on nebulized human umbilical cord MSC-derived extracellular vesicles for pulmonary fibrosis treatment. Signal Transduct. Target. Ther..

[30] Ge R., Mao Y., Zhao Y., Fan Y., Zhang Y., Tao X., Yang J. (2023). Angiotensin-converting enzyme inhibitory peptide attenuates cardiac hypertrophy associated with ACE2/Ang (1–7)/MasR axis and PKCβ1 pathway. J. Funct. Foods.

[31] Zhao Y., Cui H., Lin H., Gong W., Li N., Yang J. (2025). Angiotensin-Converting Enzyme Inhibitory Peptide Derived from Ultrafine Lycium barbarum Pomace Powder: In Vitro and In Silico Analysis. J. Agric. Food Chem..

[32] Zhou X., Xu S., Zhang Z., Tang M., Meng Z., Peng Z., Liao Y., Yang X., Nüssler A.K., Liu L. (2024). Gouqi-derived nanovesicles (GqDNVs) inhibited dexamethasone-induced muscle atrophy associating with AMPK/SIRT1/PGC1α signaling pathway. J. Nanobiotechnology.

[33] Wang Q., Liu K., Cao X., Rong W., Shi W., Yu Q., Deng W., Yu J., Xu X. (2024). Plant-derived exosomes extracted from Lycium barbarum L. loaded with isoliquiritigenin to promote spinal cord injury repair based on 3D printed bionic scaffold. Bioeng. Transl. Med..

[34] Zhang Y., Zhao B., Wang J., Zhang Z., Shen M., Ren C., Li M., Liu M., You Z., Li P. (2025). Therapeutic potential of Lycium barbarum-derived exosome-like nanovesicles in combating photodamage and enhancing skin barrier repair. Extracell. Vesicle.

[35] Shi M.M., Yang Q.Y., Monsel A., Yan J.Y., Dai C.X., Zhao J.Y., Shi G.C., Zhou M., Zhu X.M., Li S.K. (2021). Preclinical efficacy and clinical safety of clinical-grade nebulized allogenic adipose mesenchymal stromal cells-derived extracellular vesicles. J. Extracell. Vesicles.

[36] Dinh P.C., Paudel D., Brochu H., Popowski K.D., Gracieux M.C., Cores J., Huang K., Hensley M.T., Harrell E., Vandergriff A.C. (2020). Inhalation of lung spheroid cell secretome and exosomes promotes lung repair in pulmonary fibrosis. Nat. Commun..

[37] Zhu Y.G., Shi M.M., Monsel A., Dai C.-X., Dong X., Shen H., Li S.-K., Chang J., Xu C.-L., Li P. (2022). Nebulized exosomes derived from allogenic adipose tissue mesenchymal stromal cells in patients with severe COVID-19: A pilot study. Stem Cell Res. Ther..

[38] Popowski K.D., López de Juan Abad B., George A., Silkstone D., Belcher E., Chung J., Ghodsi A., Lutz H., Davenport J., Flanagan M. (2022). Inhalable exosomes outperform liposomes as mRNA and protein drug carriers to the lung. Extracell. Vesicle.

[39] Hao W., Meng H., Li H., Zheng Y., Song C., Jiang Z., Bai X., Zhang Z., Du L., Liu P. (2022). Local Application of Krill Oil Accelerates the Healing of Artificially Created Wounds in Diabetic Mice. Nutrients.

[40] Chen X., Lin H., Xiong W., Pan J., Huang S., Xu S., He S., Lei M., Chang A.C.Y., Zhang H. (2022). p53-Dependent Mitochondrial Compensation in Heart Failure with Preserved Ejection Fraction. J. Am. Heart Assoc..

[41] Fujita T., Ishikawa Y. (2011). Apoptosis in heart failure. -The role of the β-adrenergic receptor-mediated signaling pathway and p53-mediated signaling pathway in the apoptosis of cardiomyocytes. Circ. J. Off. J. Jpn. Circ. Soc..

[42] Katare P.B., Nizami H.L., Paramesha B., Dinda A.K., Banerjee S.K. (2020). Activation of toll like receptor 4 (TLR4) promotes cardiomyocyte apoptosis through SIRT2 dependent p53 deacetylation. Sci. Rep..

[43] Diepstraten S.T., Yuan Y., La Marca J.E., Young S., Chang C., Whelan L., Ross A.M., Fischer K.C., Pomilio G., Morris R. (2024). Putting the STING back into BH3-mimetic drugs for TP53-mutant blood cancers. Cancer Cell.

[44] Wei H., Qu L., Dai S., Li Y., Wang H., Feng Y., Chen X., Jiang L., Guo M., Li J. (2021). Structural insight into the molecular mechanism of p53-mediated mitochondrial apoptosis. Nat. Commun..

[45] Choe Y.J., Lee S.Y., Ko K.W., Shin S.J., Kim H.S. (2014). Nutlin-3 induces HO-1 expression by activating JNK in a transcription-independent manner of p53. Int. J. Oncol..

[46] Lee S.Y., Choi H.C., Choe Y.J., Shin S.J., Lee S.H., Kim H.S. (2014). Nutlin-3 induces BCL2A1 expression by activating ELK1 through the mitochondrial p53-ROS-ERK1/2 pathway. Int. J. Oncol..

[47] Kosiborod M.N., Deanfield J., Pratley R., Borlaug B.A., Butler J., Davies M.J., Emerson S.S., Kahn S.E., Kitzman D.W., Lingvay I. (2024). Semaglutide versus placebo in patients with heart failure and mildly reduced or preserved ejection fraction: A pooled analysis of the SELECT, FLOW, STEP-HFpEF, and STEP-HFpEF DM randomised trials. Lancet.

[48] Kintscher U., Edelmann F. (2023). The non-steroidal mineralocorticoid receptor antagonist finerenone and heart failure with preserved ejection fraction. Cardiovasc. Diabetol..

[49] Mascolo A., di Mauro G., Cappetta D., De Angelis A., Torella D., Urbanek K., Berrino L., Nicoletti G.F., Capuano A., Rossi F. (2022). Current and future therapeutic perspective in chronic heart failure. Pharmacol. Res..

[50] Doehner W., Anker S.D., Butler J., Zannad F., Filippatos G., Coats A.J., Ferreira J.P., Henrichmoeller I., Brueckmann M., Schueler E. (2024). Uric Acid and SGLT2 Inhibition with Empagliflozin in Heart Failure with Preserved Ejection Fraction: The EMPEROR-Preserved Trial. JACC Heart Fail..

[51] Taylor S.I., Blau J.E., Rother K.I. (2015). SGLT2 Inhibitors May Predispose to Ketoacidosis. J. Clin. Endocrinol. Metab..

[52] Pishdad R., Auwaerter P.G., Kalyani R.R. (2024). Diabetes, SGLT-2 Inhibitors, and Urinary Tract Infection: A Review. Curr. Diabetes Rep..

[53] Vukadinović D., Abdin A., Anker S.D., Rosano G.M., Mahfoud F., Packer M., Butler J., Böhm M. (2022). Side effects and treatment initiation barriers of sodium-glucose cotransporter 2 inhibitors in heart failure: A systematic review and meta-analysis. Eur. J. Heart Fail..

[54] Pandey A.K., Bhatt D.L., Cosentino F., Marx N., Rotstein O., Pitt B., Pandey A., Butler J., Verma S. (2022). non-steroidal mineralocorticoid receptor antagonists in cardiorenal disease. Eur. Heart J..

[55] Shunkai H., Yiting X., Shadrack S.M., Jianghao Z., Lingxiao X., Yezhi W., Fei W., Chongjiang C., Xiao X., Biao Y. (2025). Lycium barbarum (goji berry): A comprehensive review of chemical composition, bioactive compounds, health-promoting activities, and applications in functional foods and beyond. Food Chem..

[56] Li Z., Hu S., Cheng K. (2019). Chemical Engineering of Cell Therapy for Heart Diseases. Acc. Chem. Res..

[57] Xiong Y., Tang R., Xu J., Jiang W., Gong Z., Zhang L., Li X., Ning Y., Huang P., Xu J. (2022). Sequential transplantation of exosomes and mesenchymal stem cells pretreated with a combination of hypoxia and Tongxinluo efficiently facilitates cardiac repair. Stem Cell Res. Ther..

[58] Yu H., Lu K., Zhu J., Wang J. (2017). Stem cell therapy for ischemic heart diseases. Br. Med. Bull..

[59] Magadum A. (2025). Inhalable exosomes to target cardiac repair. Extracell. Vesicles Circ. Nucleic Acids.

[60] Weng H., Zou W., Tian F., Xie H., Liu A., Liu W., Liu Y., Zhou N., Cai X., Wu J. (2024). Inhalable cardiac targeting peptide modified nanomedicine prevents pressure overload heart failure in male mice. Nat. Commun..

[61] Alogna A., Berboth L., Faragli A., Ötvös J., Muzio F.P.L., di Mauro V., Modica J., Quarta E., Semmler L., Deißler P.M. (2024). Lung-to-Heart Nano-in-Micro Peptide Promotes Cardiac Recovery in a Pig Model of Chronic Heart Failure. J. Am. Coll. Cardiol..

[62] Luo H., Wang J., Lin F., Liu Y., Wu X., Li G., Su C., Chen J., Xiong F., Mo J. (2025). Macrophage exosomes mediate palmitic acid-induced metainflammation by transferring miR-3064-5p to target IκBα and activate NF-κB signaling. J. Adv. Res..

[63] Luo H., Wang K., Zhang Y., Li T., Jia X., Feng R., Zhao R., Yu R., Hu L., Zhang M. (2025). Targeted engineered exosomes alleviate myocardial infarction injury by enhancing angiogenesis and improving mitochondrial function. J. Control. Release Off. J. Control. Release Soc..

[64] Yong J., Shu H., Zhang X., Yang K., Luo G., Yu L., Li J., Huang H. (2024). Natural Products-Based Inhaled Formulations for Treating Pulmonary Diseases. Int. J. Nanomed..

[65] Takemoto Y., Kishi C., Ehira H., Matsui N., Yamaguchi T., Yoshioka Y., Matsumura S., Moriyama T., Zaima N. (2022). Inhaled turmerones can be incorporated in the organs via pathways different from oral administration and can affect weight-gain of mice. Sci. Rep..

[66] Li Z., Wang C., Xu H., Wu Q., Peng N., Zhang L., Wang H., Wu L., Li Z., Yang Q. (2025). Three-Compartment Pharmacokinetics of Inhaled and Injected Sinapine Thiocyanate Manifest Prolonged Retention and Its Therapeutics in Acute Lung Injury. Pharmaceutics.

[67] Liu C., Chen L., Ma Y., Hu K., Wu P., Pan L., Chen H., Li L., Hu H., Zhang J. (2021). Pulmonary circulation-mediated heart targeting for the prevention of heart failure by inhalation of intrinsically bioactive nanoparticles. Theranostics.

[68] Miragoli M., Ceriotti P., Iafisco M., Vacchiano M., Salvarani N., Alogna A., Carullo P., Ramirez-Rodríguez G.B., Patrício T., Degli Esposti L. (2018). Inhalation of peptide-loaded nanoparticles improves heart failure. Sci. Transl. Med..

[69] Li J., Minćzuk K., Massey J.C., Howell N.L., Roy R.J., Paul S., Patrie J.T., Kramer C.M., Epstein F.H., Carey R.M. (2020). Metformin Improves Cardiac Metabolism and Function, and Prevents Left Ventricular Hypertrophy in Spontaneously Hypertensive Rats. J. Am. Heart Assoc..

[70] Kim J., Zhang X., Wang R., Najer A., Lau Q.Y., Cammack-Najera A., Kim J.A., Kang Y.K., Xie R., Kim H. (2025). Vascularized and Perfusable Human Heart-on-a-Chip Model Recapitulates Aspects of Myocardial Ischemia and Enables Analysis of Nanomedicine Delivery. Adv. Mater..

[71] Zhu K., Wang K., Zhang R., Zhu Z., Wang W., Yang B., Zhao J., Shen Y. (2025). Iron chelators loaded on myocardiocyte mitochondria-targeted nanozyme system for treating myocardial ischemia-reperfusion injury in mouse models. J. Nanobiotechnology.

[72] Mashele S.S. (2025). Stimuli-Responsive, Cell-Mediated Drug Delivery Systems: Engineering Smart Cellular Vehicles for Precision Therapeutics. Pharmaceutics.

[73] Zhen P., Jiang Q., Yu F., Xu X., Wei Q., Liu X., Sun X., Liang G., Tong J. (2025). ROS-differentiated release of Apelin-13 from hydrogel comprehensively treats myocardial ischemia-reperfusion injury. J. Control. Release Off. J. Control. Release Soc..

[74] Schiattarella G.G., Altamirano F., Tong D., French K.M., Villalobos E., Kim S.Y., Luo X., Jiang N., May H.I., Wang Z.V. (2019). Nitrosative stress drives heart failure with preserved ejection fraction. Nature.

