# Supplementary material for: Lycium barbarum-Derived Extracellular Vesicles Ameliorate Myocardial Hypertrophy in Heart Failure with Preserved Ejection Fraction, Associated with Reduced Cardiomyocyte Apoptosis
Source: Nutrients. 2026 Aug 5;18(15):2564. doi: 10.3390/nu18152564 (PMC13468228; doi:10.3390/nu18152564)
Supplement: Supplementary file 1 [file nutrients-18-02564-s001.zip › nutrients-4441550-supplementary.pdf]

Supplemental materials

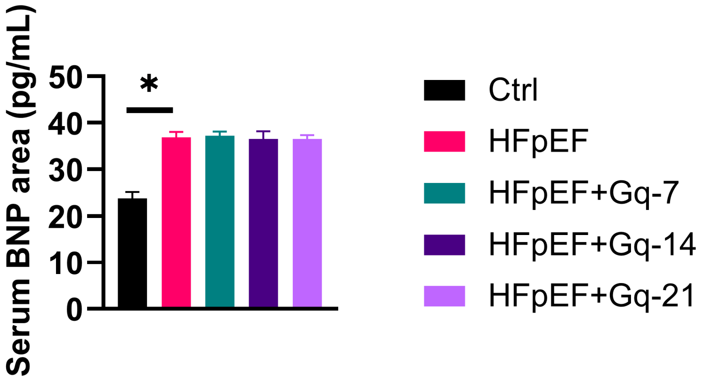

Figure S1. Serum BNP after 8 weeks of induction. The key validation data for the HFpEF model - serum BNP data to confirm successful establishment of the HFpEF phenotype after 8 weeks of induction. The data were summarized as means ± SD. *, $P < 0.05$ vs. Ctrl.

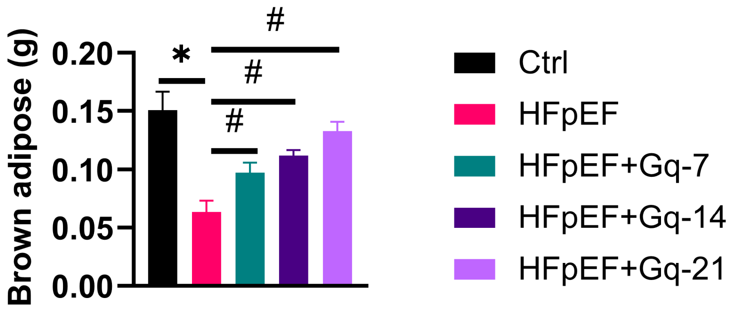

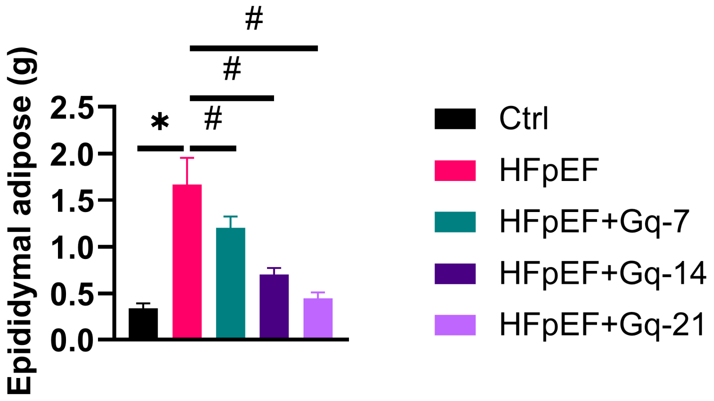

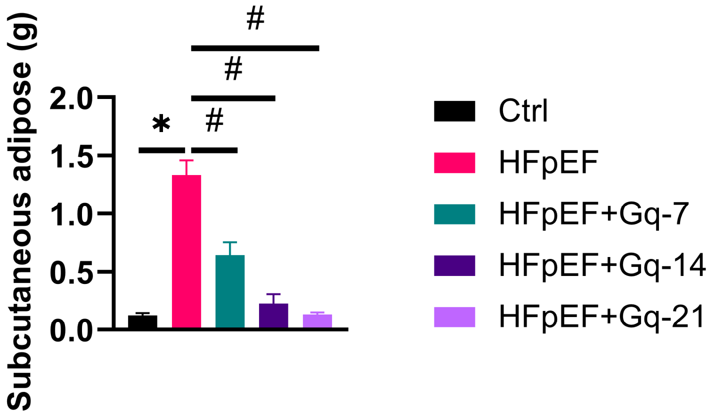

Figure S2. Statistical analysis of the weights of murine brown adipose, subcutaneous adipose and epididymal adipose. The data were summarized as means ± SD. *, $P < 0.05$ vs. Ctrl; #, $P < 0.05$ vs HFpEF.

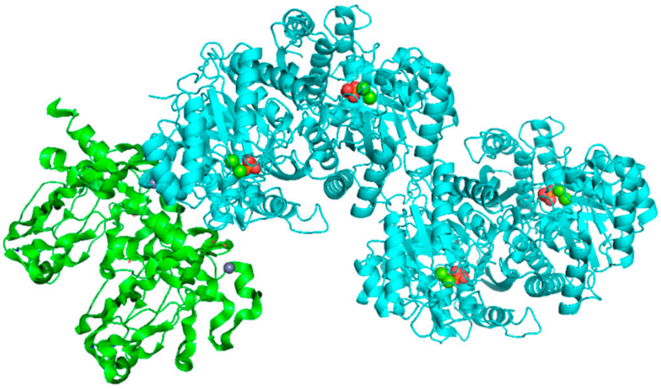

Rank	
Docking Score	-264.38
Confidence Score	0.9078

Molecular docking of miRNA in Gq-EVs: ata-miR156c-3p and TP53

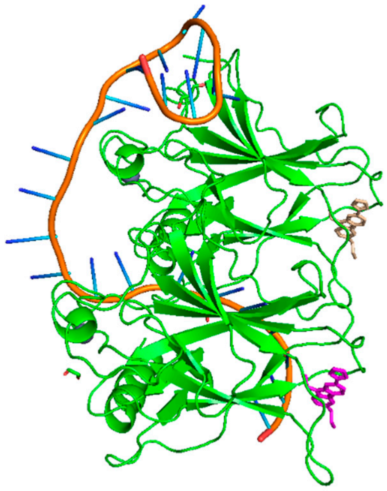

Rank	
Docking Score	-333.24
Confidence Score	0.9750

Molecular docking of the proteins with the highest abundance in Gq-EVs: Enolase1 and TP53

Figure S3. Evidence for TP53 as a core target. Gq-EVs carry TP53 modulating miRNAs ata-miR156c-3p and proteins Enolase1. The total docking scores were-264.38 and-333.24, indicating that ata-miR156c-3p and Enolase1 exhibit extremely strong binding affinity to TP53 with highly

stable conformations (values ≤-200 signify strongly stable binding, corresponding to a naturally credible binding pattern). The Confidence Scores of 0.9078 and 0.9750 suggest that the docked conformations are highly likely to approximate the native structure (range: 0~1; ≥0.7 indicates highly reliable binding with exceptional model credibility).

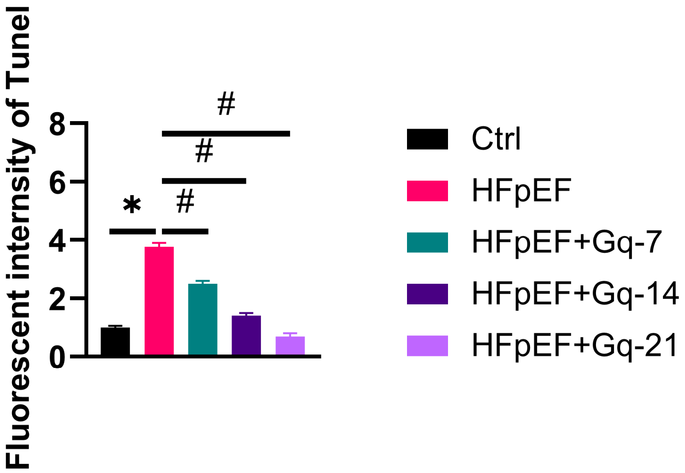

Figure S4.Quantitative analysis for TUNEL staining. The data were summarized as means ± SD. *, $P < 0.05$ vs. Ctrl; #, $P < 0.05$ vs HFpEF.

Table S1 Primer sequences

**Genes**	**The primers (M)**
β-Mhc-forward	CCACAACCAGTACAAGTTCG
β-Mhc-reverse	CTGGATGATCAGCAAGGAGT
Bnp-forward	CTGAAGGTGCTCCCAGATGATTC
Bnp-reverse	GACTTCCCAGAGGATAGGAGTGACC
Tp53-forward	AGAGACCGCCGTACAGAAGA
Tp53-reverse	GCATGGGCATCCTTTAACTC

**Genes**	**The primers (H)**
BAX-forward	TCCTGCACCTGTTACGGTTT
BAX-reverse	TGCTTCCAATTACTTTAATCCTTTTT
BCL2-forward	CGCCCCTGCTATGGTTTAG
BCL2-reverse	GAGATTTCAGCTGCTCTTGGAC
DRP1-forward	AAGCTGCTGCCATAGTCCTC
DRP1-reverse	ACCACAGCCATGTCAGTGTC
β-MHC-forward	GGGCAAAGGCAAGGCCAAGAAA
β-MHC-reverse	ATGGGTGGAGCGCAAGTTGGTCA
BNP-forward	TGGAAACGTCCGGGTTACAGGA
BNP-reverse	TCCGGTCCATCTTCCTCCCAAA
MFN2-forward	CCGCCACATAGAGGAAGGAC
MFN2-reverse	CGCACAGACACAGGAAGGAG
OPA1-forward	CGACCCCAATTAAGGACATCC

OPA1-reverse	GCGAGGCTGGTAGCCATATTT
PGC-1α-forward	GTCACCACCCAAATCCTTAT
PGC-1α-reverse	ATCTACTGCCTGGAGACCTT
TP53-forward	GAGCGAATCACGAGGGAC
TP53-reverse	GCACAAACACGGACAGGA
